# Supplementary material for: Manipulating mechanical strength of isoreticular two-dimensional polyamide materials via multiple interactions
Source: Nat Commun. 2025 Nov 23;16:11580. doi: 10.1038/s41467-025-66696-7 (PMC12748856; doi:10.1038/s41467-025-66696-7)
Supplement: Supplementary file 1 — Supplementary information [file 41467_2025_66696_MOESM1_ESM.pdf]

Supplementary Information

Manipulating mechanical strength of isoreticular two-dimensional polyamide materials via multiple interactions

Qing Hu[1], Chiran Wang[1], Yuan Zhao[1], Awei Hu[2] and Bo Liu[1,2]*

[1]School of Chemistry and Materials Science, University of Science and Technology of China, Hefei, Anhui 230026 (China).

[2]Hefei National Research Center for Physical Sciences at the Microscale, University of Science and Technology of China, Hefei, Anhui 230026 (China).

*Corresponding author. Email: liuchem@ustc.edu.cn (B.L.)

Table of Contents

Section 1. Material characterization

Supplementary Fig. 1. Synthesis scheme and reaction process of GH-TMC.
Supplementary Fig. 2. Mass spectrometry of GH-TMC.
Supplementary Fig. 3. Solid state ^{13}C NMR spectra of GH-TMC.
Supplementary Fig. 4. XPS spectra of GH-TMC.
Supplementary Fig. 5. N_2 adsorption-desorption isotherms for GH-TMC.
Supplementary Fig. 6. P-XRD and FTIR spectra of Melem.
Supplementary Fig. 7. XPS spectra of Melem, Melem-TPC and Melem-TMC.
Supplementary Fig. 8. FTIR and TGA curves of Melem-TPC and Melem-TMC.
Supplementary Fig. 9. FTIR spectra of GH-TMC obtained at different reaction times
Supplementary Fig. 10. NMR of GH-TMC at different concentrations.
Supplementary Fig. 11. Raman spectrum of GH-TMC.
Supplementary Fig. 12. Determination of average H-bond numbers using FTIR.
Supplementary Fig. 13. Characterization of Melem-TMC nanosheets.
Supplementary Fig. 14. Characterization of GH-TPC nanosheets.
Supplementary Fig. 15. Characterization of GH-OC nanosheets.
Supplementary Fig. 16. SEM mapping result of GH-TMC sample.
Supplementary Fig. 17. Selected electron diffraction and TEM images of GH-TMC.
Supplementary Fig. 18. TEM images of a series of 2D polymers.
Supplementary Fig. 19. Top-view SEM images of a series of 2D polymer films.
Supplementary Fig. 20. AFM topology of spin-coated GH-TMC film.
Supplementary Fig. 21. Cross-sectional SEM images of GH-TMC films.
Supplementary Fig. 22. Plot of nanofilm thickness against dispersion concentration.
Supplementary Fig. 23. AFM topology of ultra-thin spin-coated GH-TMC film.
Supplementary Fig. 24. Young's modulus distribution of a series of 2D polymer films.
Supplementary Fig. 25. Schematic diagram of the GH-TMC structure.
Supplementary Fig. 26. FTIR spectra of amide and imide 2D polymers.
Supplementary Fig. 27. MD simulates the dynamic changes of H-bonds in GH-TMC.
Supplementary Fig. 28. Mechanical properties of GH-TMC.
Supplementary Fig. 29. Indentation process at 120 nm indentation depth.
Supplementary Fig. 30. Stiffness and strength of GH-TMC film in different regions.
Supplementary Fig. 31. Indentation process at 700 nm indentation depth.
Supplementary Fig. 32. Representative load-displacement curves of GH-TMC film.
Supplementary Table 1. Young's modulus of a series of 2D polymer films.
Supplementary Table 2. Summary of Young's modulus for other countertypes.
Supplementary Table 3. Summary of *We* and *H* of other countertypes.
Supplementary Table 4. Comparison between the H^3/E^2 versus H of porous films.
Supplementary Table 5. Comparison between the H^3/E^2 versus H of polymers.
Supplementary Table 6. Comparison between the H^3/E^2 versus H of Metals.
Supplementary Note 1. The specific influence of multiple interactions on the mechanical properties of GH-TMC films.

Section 2. Supplementary References

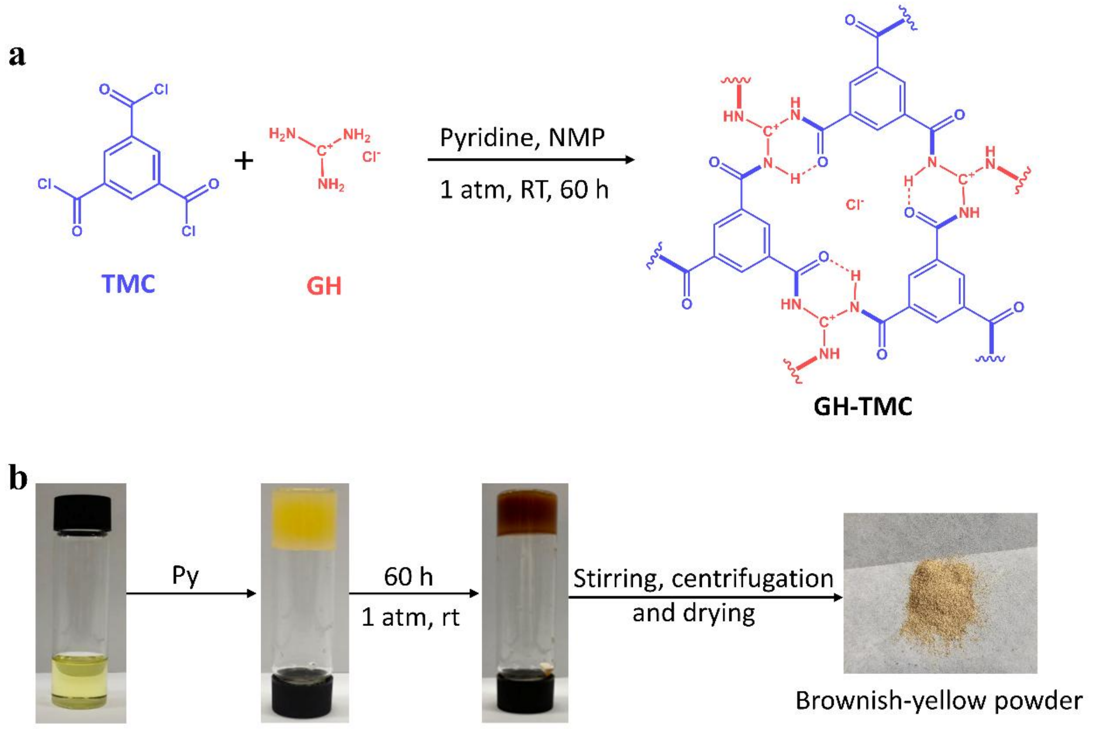

Supplementary Fig. 1. Synthesis scheme and reaction process of GH-TMC. a, Schematic diagram of the synthesis scheme of GH-TMC. **b**, Reaction and purification process of GH-TMC.

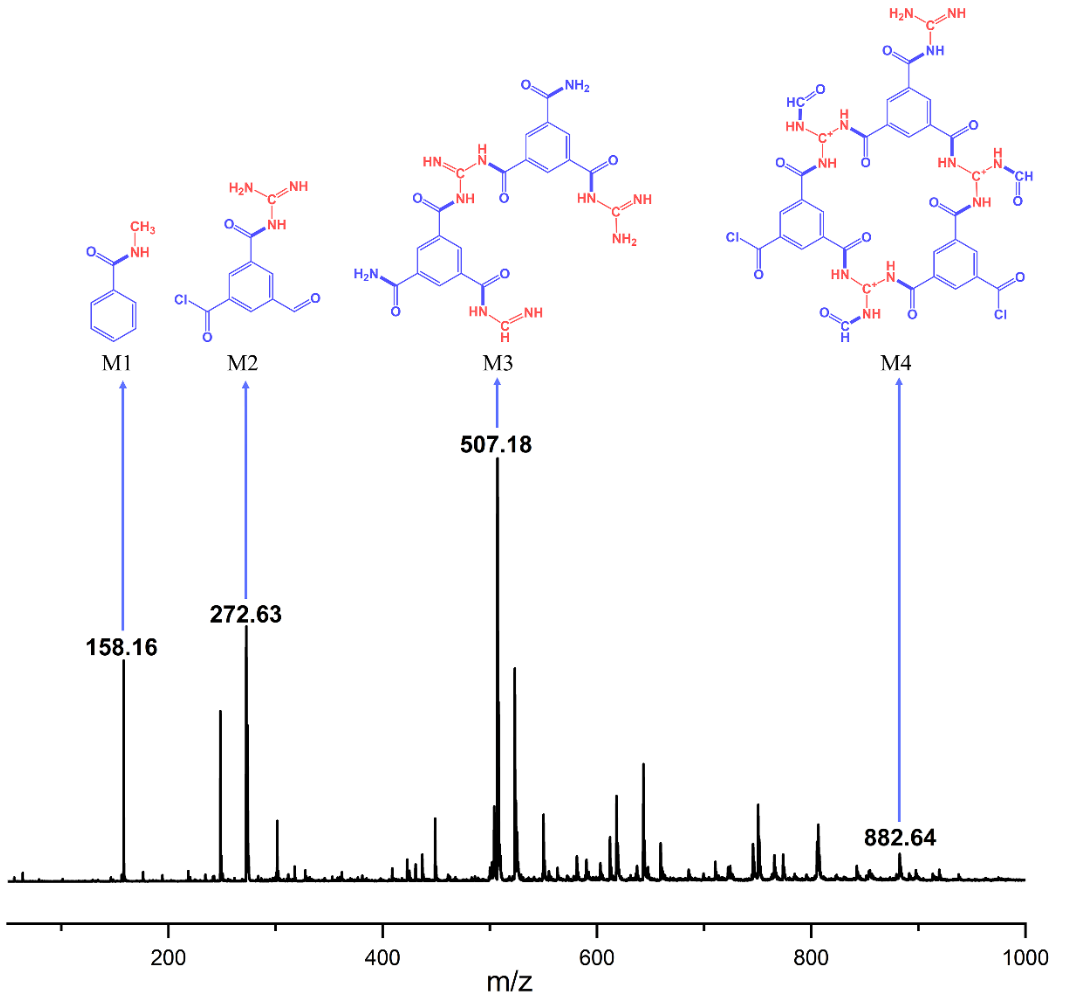

Supplementary Fig. 2. Mass spectrometry of GH-TMC. Four main molecular fragments with the highest intensity in the mass spectrum are labeled as M1, M2, M3 and M4. From M4 with the highest m/z, it can be clearly found that the basic structural unit of GH-TMC is a six-membered ring connected by three GH molecules and TMC molecules through amide bonds.

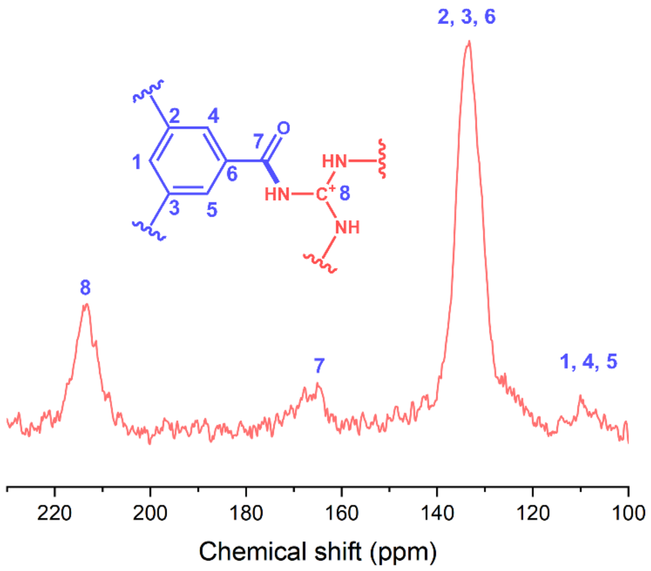

Supplementary Fig. 3. Solid-state ^{13}C NMR spectrus of GH-TMC (inset represents the repeating fragment). The chemical shift at the center of ca. 133 ppm is assigned to aromatic C, the peak around 166 ppm is attributed to the C atoms in the amide, and the signal at 213 ppm is from C^+ in guanidium motif.

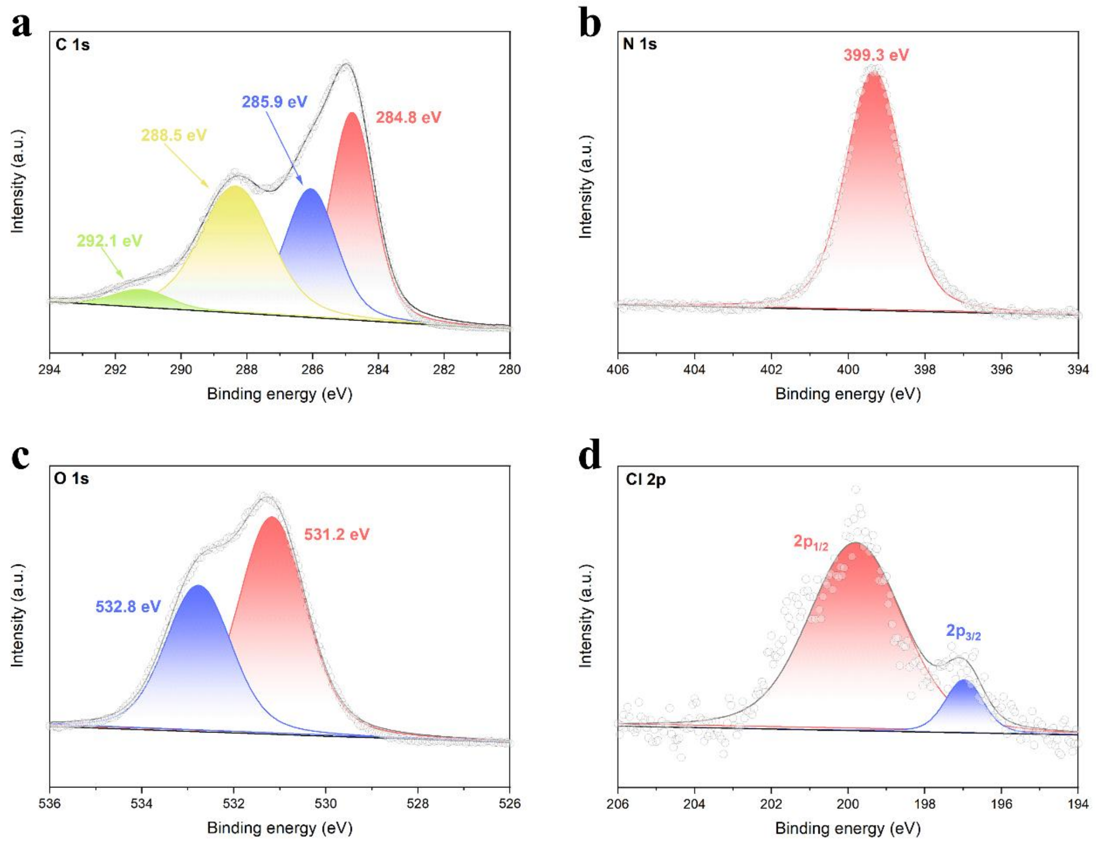

Supplementary Fig. 4. XPS spectra of GH-TMC. a, C 1*s*. **b**, N 1*s*. **c**, O 1*s*. **d**, Cl 2*p*. The XPS spectral results of GH-TMC reveal the environment of chemical bonding. In the C 1*s* spectrum, the GH-TMC is deconvoluted into four peaks of 284.8 eV, 285. eV, 288.5 eV and 292.1 eV, respectively. Among them, the peak at 284.8 eV is standard C, and the peak at 285.9 eV and 288.5 eV represents the C atom in the benzene ring and amide (-NH-C=O), respectively. Note that the peak with the highest electron binding energy at 292.1 eV is attributed to the C^+ ion in the guanidyl group, which means that the GH-TMC molecules prefer the AB stacking structure. The N 1*s* spectrum has only one peak of 399.3 eV, which is attributed to the N atom in the amide (-NH-C=O). This means that the synthesized GH-TMC molecule has fewer defects. In addition, in the O 1*s* spectrum, two deconvolved peaks of 531.2 eV and 532.8 eV represent the O atoms in -OH and amide (-NH-C=O), respectively. The combination of the C 1*s*, N 1*s* and O 1*s* spectra demonstrates the formation of amide bonds as a key connecting node in the GH-TMC molecule, which is consistent with the results of FTIR spectroscopy. The XPS spectra of Cl 2*p* indicate that Cl^- ions are distributed in GH-TMC molecules as counter anions to C^+ ions.

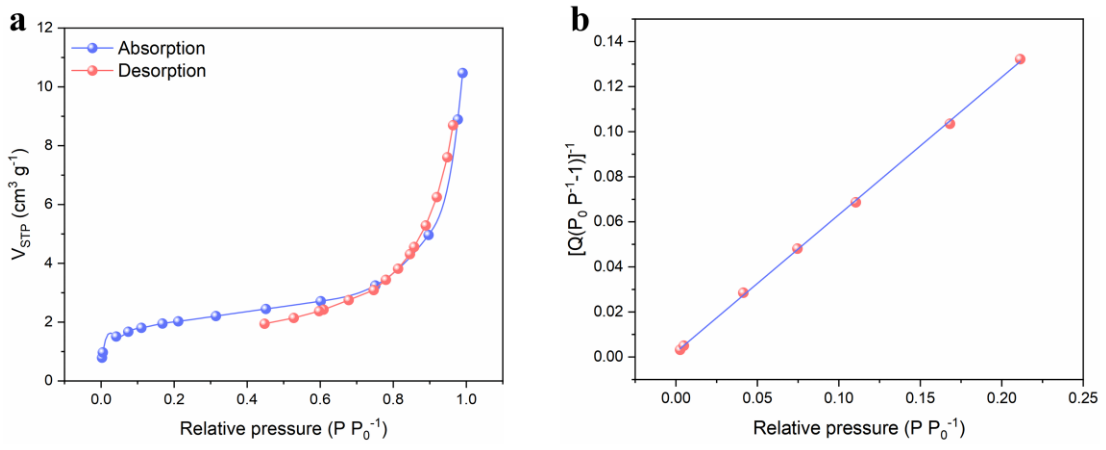

Supplementary Fig. 5. N_2 adsorption-desorption isotherms for GH-TMC. a, N_2 adsorption-desorption isotherms for GH-TMC. **b**, BET plot for N_2 sorption in GH-TMC. It is important for Brunauer-Emmett-Teller (BET) analysis to help in validating the proposed porous structure. The BET surface area is measured by using a BEL sorp-max machine, BEL, Japan and then calculated from N_2 sorption at 77 K over the pressure range 0.01-0.02 P P_0^{-1}. The synthesized GH-TMC sample was first heated and vacuumed at 120 °C for degassing, and then N_2 adsorption was carried out. The adsorption isotherm of GH-TMC is shown in Supplementary Fig. 5, which is a typical type II curve, associating with nanoparticles-stacking structure. At relatively low pressures, the adsorption isotherm increases rapidly, mainly due to monolayer adsorption. Then it increases slowly, and finally it increases exponentially, mainly due to multi-layer adsorption. The specific surface area calculated by the adsorption isotherm is 7.12 m^2 g^{-1}. This result indicates that bulk materials have very little accessible free volume or inner surface, and stacking does not cause the pores or rings within the molecules to be neatly arranged. Each layer of GH-TMC nanosheets is arranged in an AB interlaced manner. This is consistent with the proposed structural mode of GH-TMC.

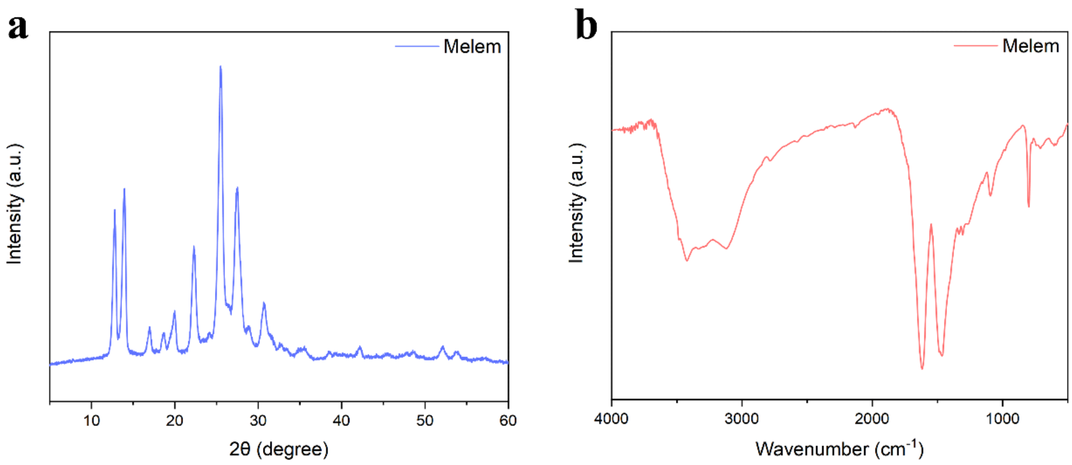

Supplementary Fig. 6. PXRD and FTIR spectra of Melem. a, PXRD of Melem. **b**, FTIR spectra of Melem.

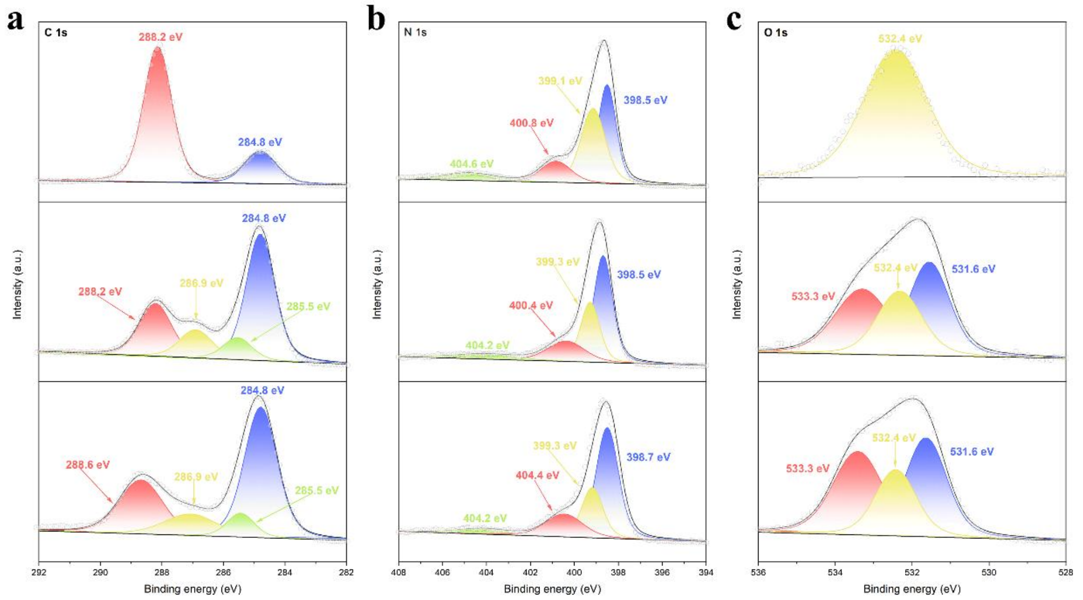

Supplementary Fig. 7. XPS spectra of Melem, Melem-TPC and Melem-TMC. a, C 1*s*. **b**, N 1*s*. **c**, O 1*s*. (From top to bottom are Melem, Melem-TPC and Melem-TMC). In the C 1*s* spectrum, the two deconvoluted peaks of Melem at 284.8 eV and 288.2 eV represent the standard C and the C atom in the triazine ring (N-C=N), respectively. The remaining two peaks of 285.5 eV and 286.9 eV in Melem-TPC and Melem-TMC originate from C atoms in the benzene ring and the amide (-NH-C=O), respectively. The four convoluted peaks in the N1*s* spectrum all originate from N atoms in different chemical environments in Melem. Note that the peak of 399.3 eV in Melem-TPC and Melem-TMC is attributed to the N atom in the amide (-NH-C=O). In the O 1*s* spectrum, the peaks of 531.6 eV and 533.3 eV represent the O atoms in -OH and the amide (-NH-C=O), respectively.

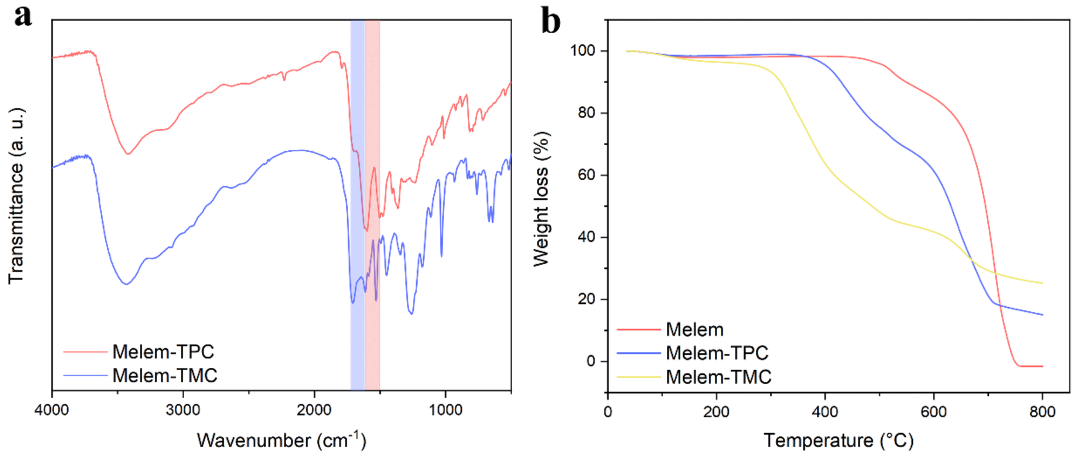

Supplementary Fig. 8. FTIR spectra and thermogravimetric profile of Melem, Melem-TPC and Melem-TMC. a, FTIR spectra of Melem TPC and Melem-TMC. **b**, Thermogravimetric profile of Melem, Melem-TPC and Melem-TMC. The peaks in the blue region (amide I) and the red region (amide II) in the FTIR spectrum confirm the formation of amide bonds, which is consistent with the previous XPS results. This shows that the 2D polymer synthesized with Melem as a monomer is similar to GH-TMC, with amide bonds as connecting nodes. For TGA measurements, both samples were tested under a N_2 atmosphere with a heating rate of 10 °C min^{-1} and a temperature range of 30 °C-800 °C.

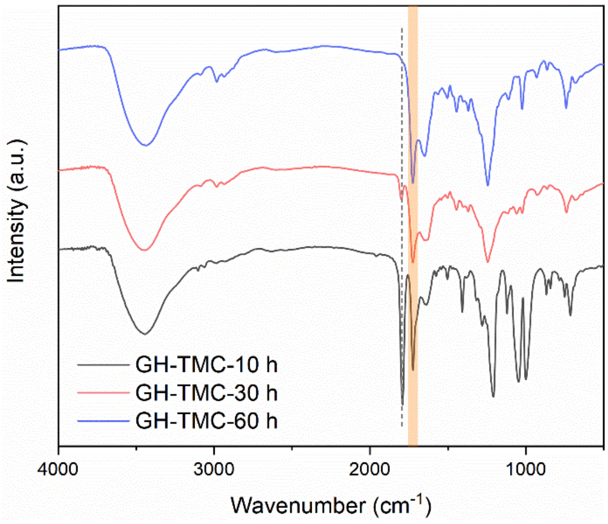

Supplementary Fig. 9. FTIR spectra of GH-TMC obtained at different reaction times: 10 h, 30 h, and 60 h.

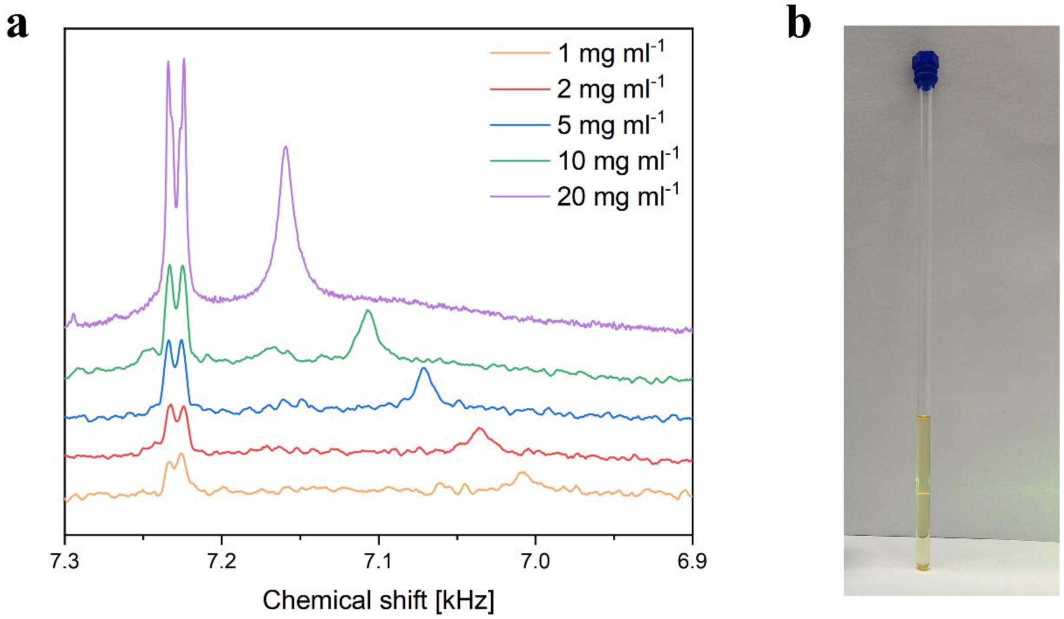

Supplementary Fig. 10. Segments of single-pulse spectra of GH-TMC dissolved in dimethyl sulfoxide (DMSOD6) at different concentrations. a, Segments of single-pulse spectra of GH-TMC at different concentrations. **b**, Digital photo of 20 mg mL^{-1} GH-TMC dispersion with weak Tyndall effect. The spectrum was obtained using a 600 MHz Superconducting Fourier Nuclear Magnetic Resonance Spectrometer operating at 14.1T and 32 scans were collected. Where yellow, red, blue, green and purple represent the concentration of 1, 2, 5, 10 and 20 mg mL^{-1} GH-TMC respectively. As the concentration increases, the amide protons shift downfield, indicating their participation in intermolecular H-bonding.

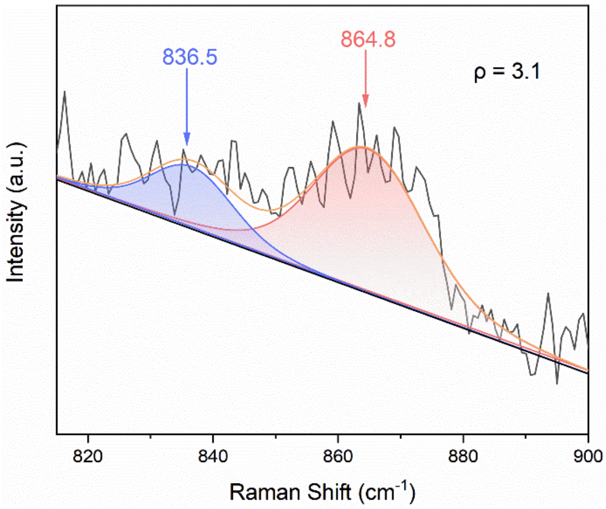

Supplementary Fig. 11. Raman spectrum of GH-TMC. The Raman spectra of GH-TMC show double peaks between 830 cm^{-1} and 860 cm^{-1}, and the ratio ρ=3.1 is much higher than 1, which indicates that GH-TMC has strong hydrogen bond interaction.

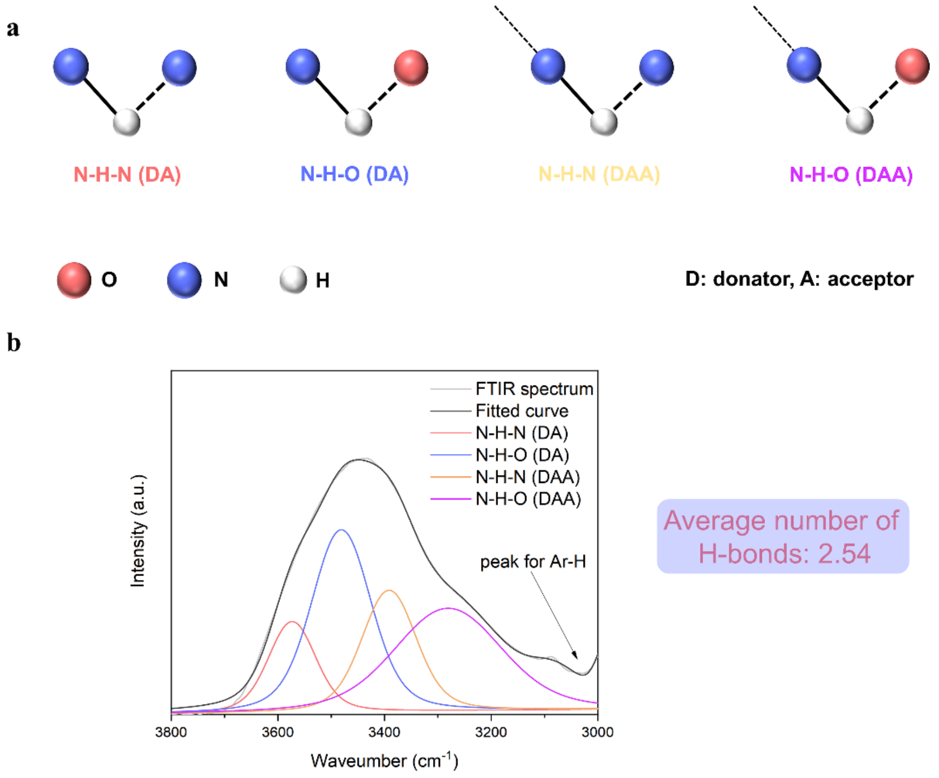

Fitted peak	N-H-N (DA)	N-H-O (DA)	N-H-N (DAA)	N-H-O (DAA)
Wavenumber (cm^{-1})	3573 cm^{-1}	3480 cm^{-1}	3391 cm^{-1}	3282 cm^{-1}

Supplementary Fig. 12. Determination of average H-bond numbers using infrared spectroscopy. a, Four H-bond configurations of GH-TMC. **b**, FTIR spectra of GH-TMC. According to previous experimental and computational studies on H_2O molecules, it was proved that there are five H-bond configurations[1]. By similar methods, we also analyzed four different H-bond configurations in GH-TMC, including N-H-N (DA, ~3573 cm^{-1}) that donates and accepts one H-bond, N-H-O (DA, ~3480 cm^{-1}) that donates and accepts one H-bond, N-H-N (DAA, ~3391 cm^{-1}) that donates one and accepts two H-bonds and N-H-O (DAA, ~3282 cm^{-1}) that donates one and accepts two H-bonds. The average number of H-bonds in GH-TMC was estimated to be ~2.54, which aligned closely with high-performance aramids like Kevlar (~2)[2] and significantly exceeds typical nylons (1.3-1.8)[3]. This indicates the presence of exceptionally strong intramolecular H-bonds on the GH-TMC, contributing to its higher resistance to external impacts. Consequently, this characteristic enhances its Young's modulus, making it mechanically robust.

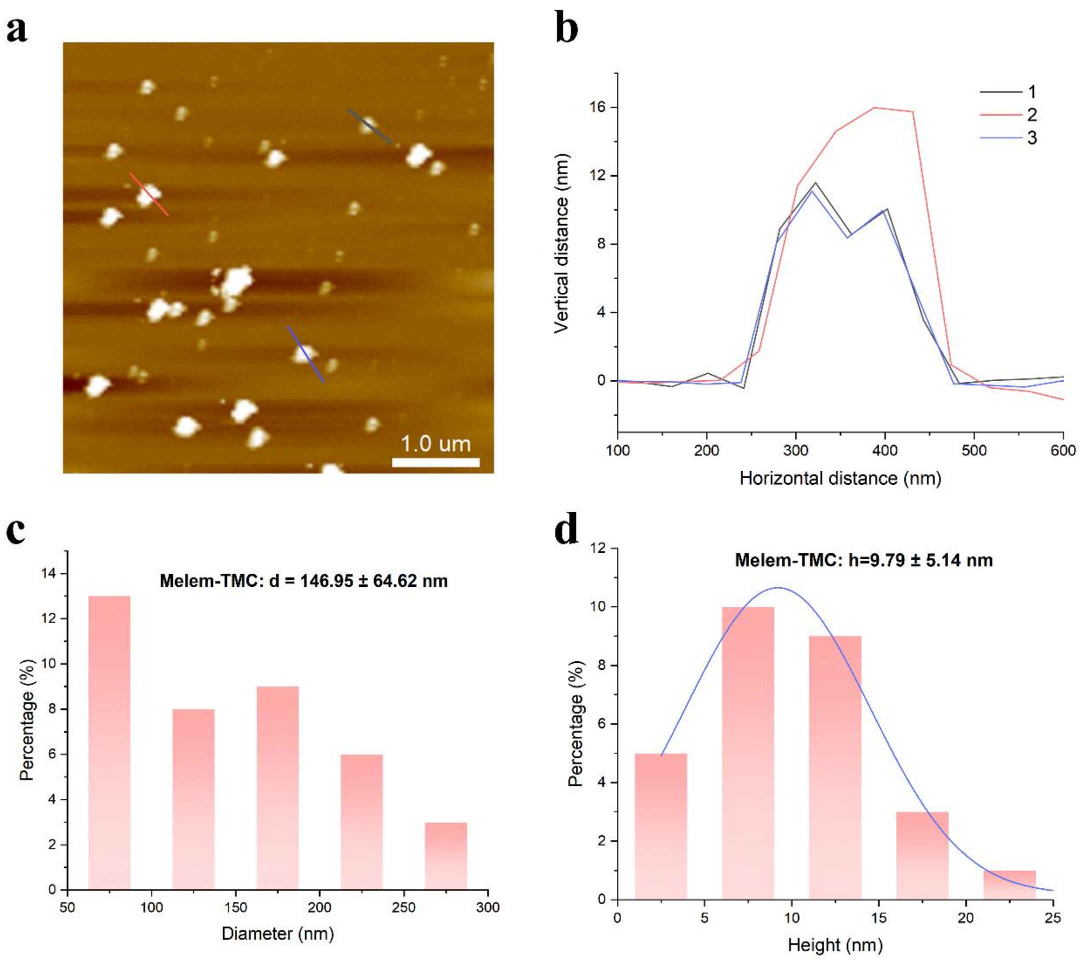

Supplementary Fig. 13. Characterization of Melem-TMC nanosheets. a, AFM image of Melem-TMC nanosheets. **b**, Height profiles along the different colored lines indicated in a (from top to bottom: black, red and blue). **c**, **d**, Size and height distribution of observed Melem-TMC nanosheets respectively. Measurements of lateral dimensions and thickness reveal an average lateral size of 146.95 ± 64.62 nm and an average thickness of 9.79 ± 5.14 nm.

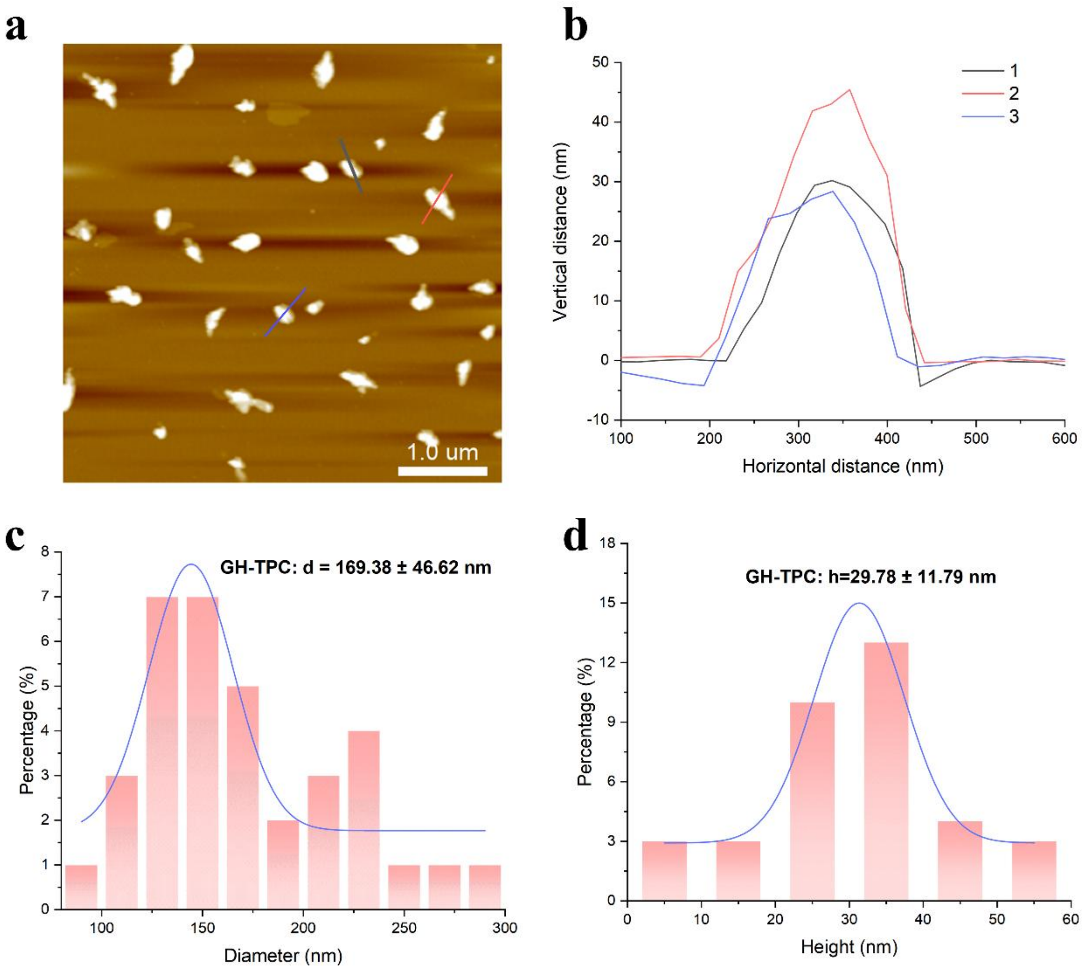

Supplementary Fig. 14. Characterization of GH-TPC nanosheets. a, AFM image of GH-TPC nanosheets. **b**, Height profiles along the different colored lines indicated in a (from top to bottom: black, red and blue). **c**, **d**, Size and height distribution of observed GH-TPC nanosheets respectively. Measurements of lateral dimensions and thickness reveal an average lateral size of 169.38 ± 46.62 nm and an average thickness of 29.78 ± 11.79 nm.

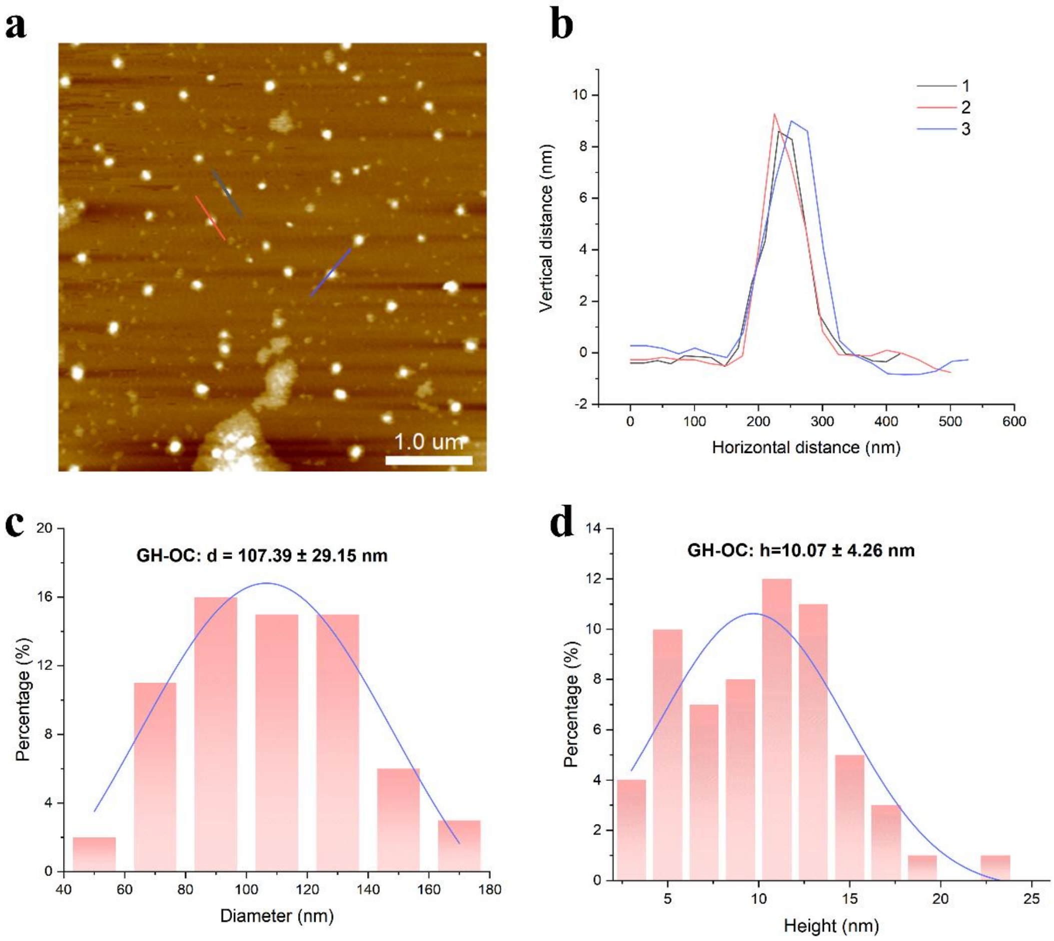

Supplementary Fig. 15. Characterization of GH-OC nanosheets. a, AFM image of GH-OC nanosheets. **b**, Height profiles along the different colored lines indicated in a (from top to bottom: black, red and blue). **c**, **d**, Size and height distribution of observed GH-OC nanosheets respectively. Measurements of lateral dimensions and thickness reveal an average lateral size of 107.39 ± 29.15 nm and an average thickness of 10.07 ± 4.26 nm.

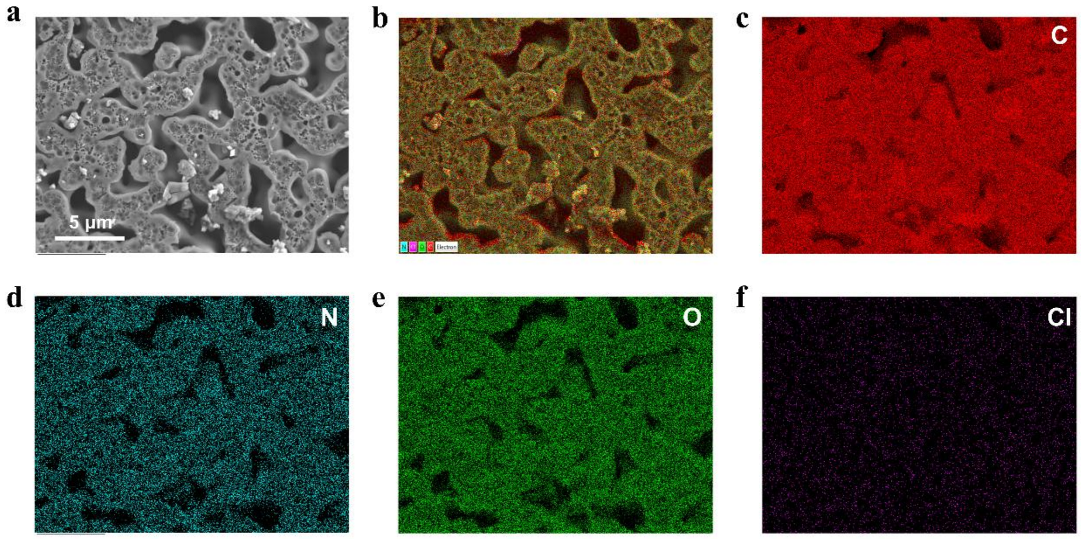

Supplementary Fig. 16. SEM mapping result of GH-TMC sample. a, **b**, SEM image and EDS image of GH-TMC sample, respectively (scale bar: 5 μm). **c-f**, Elemental mapping results. **c**, C. **d**, N. **e**, O. **f**, Cl.

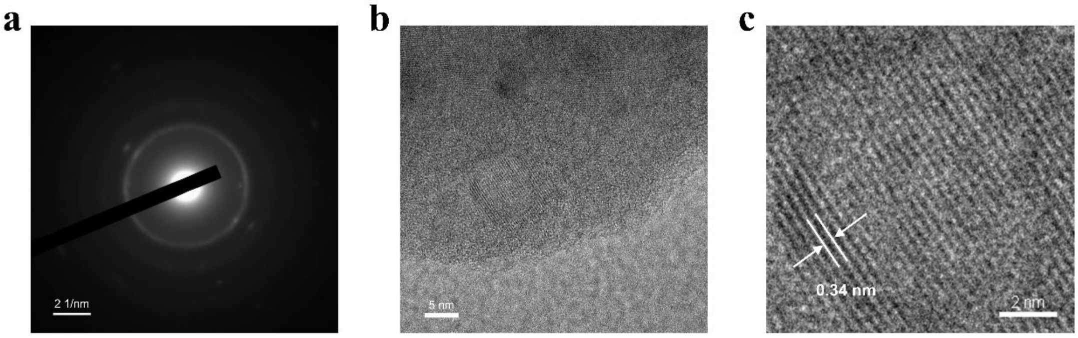

Supplementary Fig. 17. Selected electron diffraction and HR-TEM images of GH-TMC. a, Selected electron diffraction image of GH-TMC. **b**, **c**, HR-TEM images of GH-TMC. A diffraction ring appears in the selected electron diffraction image of GH-TMC, corresponding to the layer spacing of 0.34 nm. Accordingly, HR-TEM images also show clear lattice fringes with a spacing of 0.34 nm. This is consistent with the results of P-XRD, WAXS and simulation.

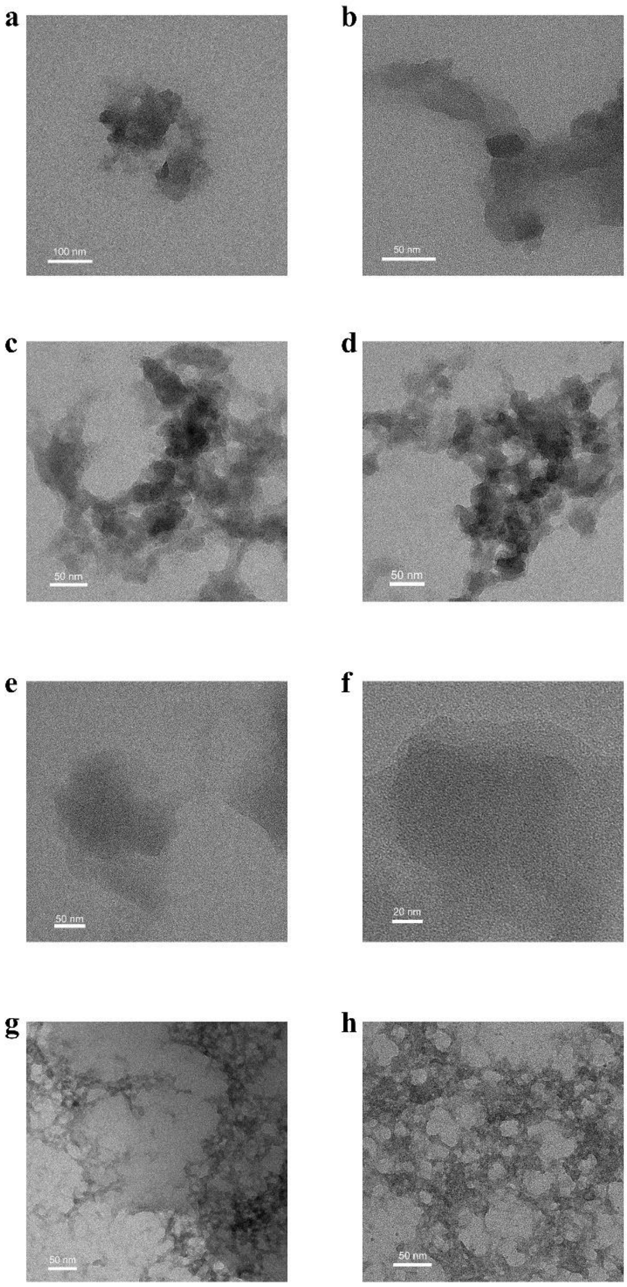

Supplementary Fig. 18. TEM images of a series of 2D polymers. a, **b**, Melem-TPC. **c, d**, Melem-TMC. **e**, **f**, GH-TPC. **g**, **h**, GH-OC. Scale bars, 100 nm (**a**), 50 nm (**b**, **c**, **d**, **e**, **g**, **h**), 20 nm (**f**). All synthesized 2D polymers exhibited distinct lamellar features.

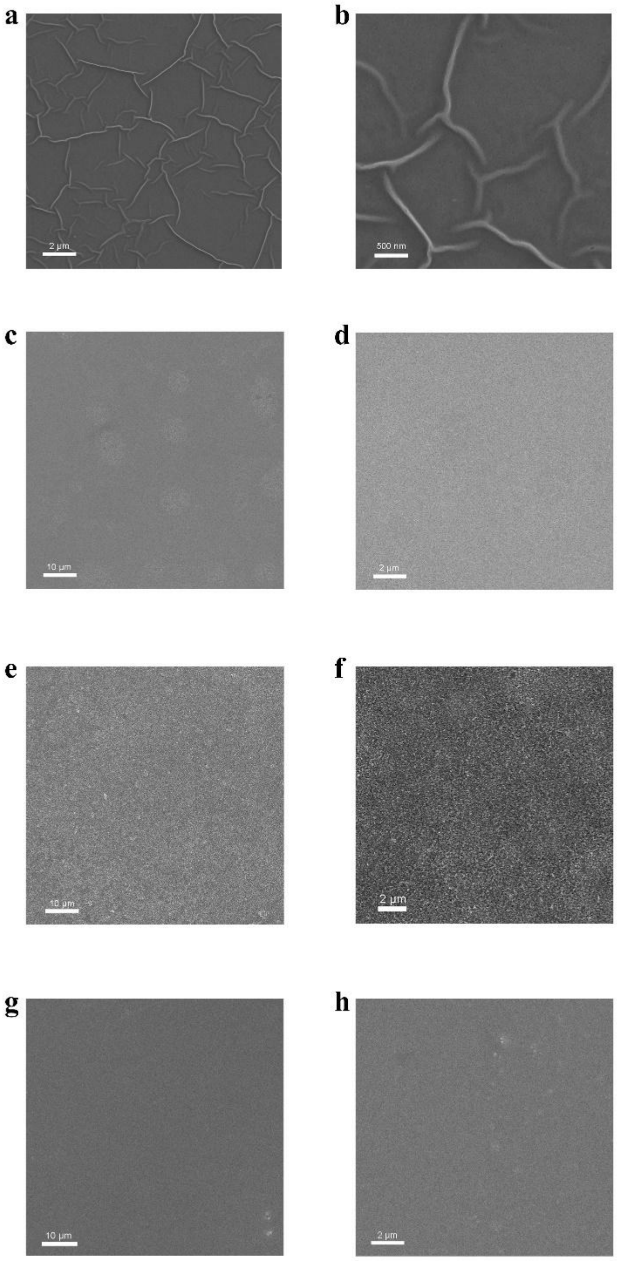

Supplementary Fig. 19. Top-view SEM images of a series of 2D polymer films. a, **b**, Melem-TPC. **c**, **d**, Melem-TMC. **e**, **f**, GH-TPC. **g**, **h**, GH-OC. Scale bars, 500 nm (**b**), 2 μm (**a**, **d**, **f**, **h**), 10 μm (**c**, **e**, **g**). All of these spin-coated films have flat surfaces with no obvious cracks and pinholes.

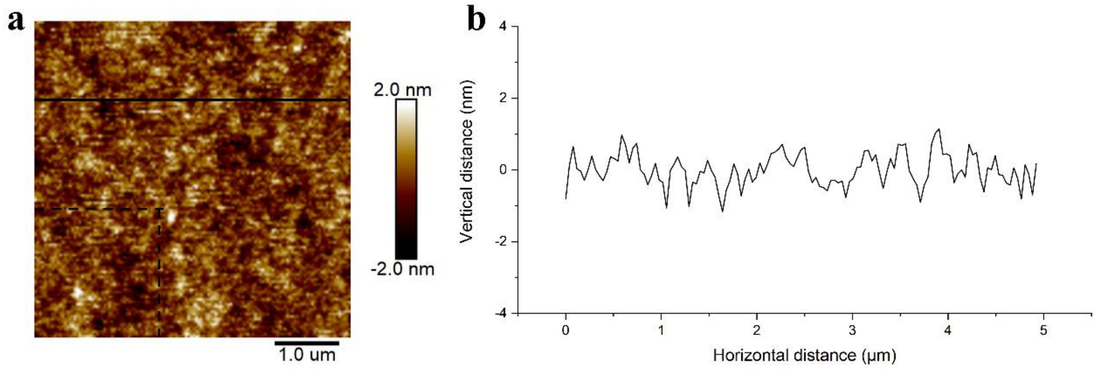

Image Size	Calculated roughness
5 * 5 μm (from a)	690 pm
2 * 2 μm (black square in a)	529 pm

Supplementary Fig. 20. AFM topology of spin-coated GH-TMC film. a, AFM image of GH-TMC film. **b**, Corresponding height profile along the black line in (**a**). All of these films with spin coatings have hyperplanar surfaces. Their roughness is usually in the region of 5 * 5 μm and ranges from 500-700 pm, similar to commercial ultra-flat silicon wafers. The HR-AFM characterization of thin films also provides some topological information. However, unlike measurements of individual nanosheets on a silicon wafer substrate, the height picture of the film is blurry, likely due to the softness of the 2D molecules and their disproportionate (random) stacking.

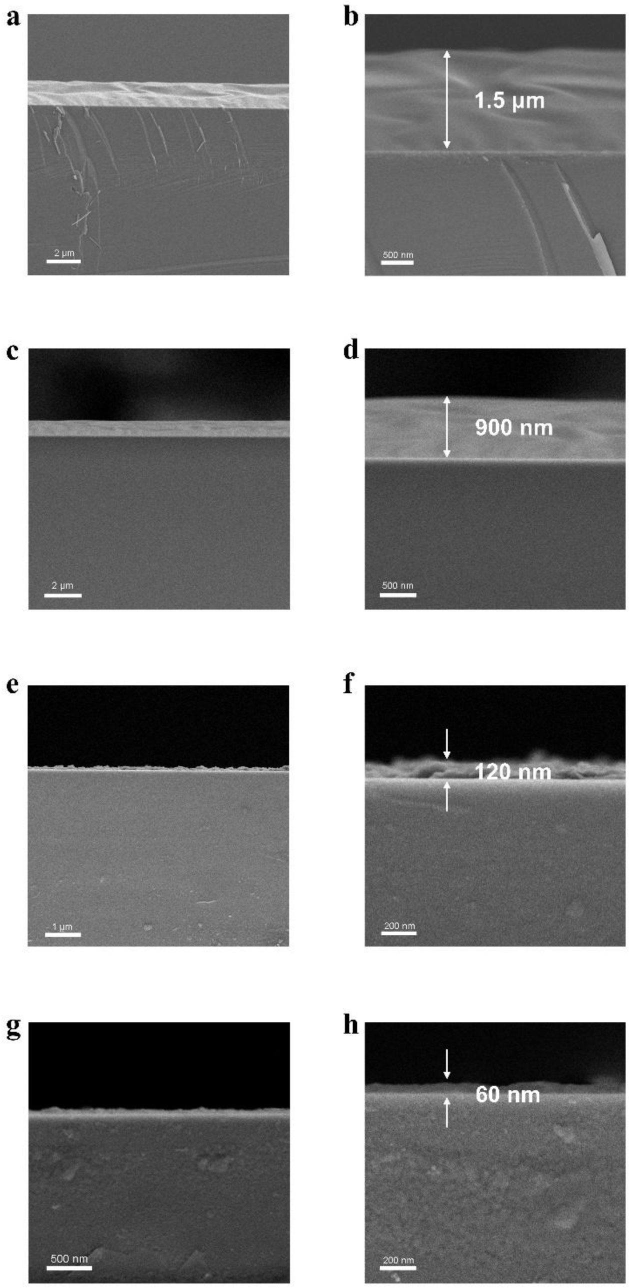

Supplementary Fig. 21. Cross-sectional SEM images of GH-TMC films with different thicknesses. a, **b**, 10 mg mL^{-1}. **c**, **d**, 5 mg mL^{-1}. **e**, **f**, 1 mg mL^{-1}. **g**, **h**, 0.5 mg mL^{-1}. Scale bars, 2 μm (**a**, **c**), 1 μm (**e**), 500 nm (**b**, **d**, **g**), 200 nm (**f**, **h**). SEM cross-section images showed the thickness of films produced by spin-coating with concentrations of 10 mg mL^{-1}, 5 mg mL^{-1}, 1 mg mL^{-1} and 0.5 mg mL^{-1} GH-TMC dispersions, which were about 1.5 μm, 900 nm, 120 nm and 60 nm, respectively.

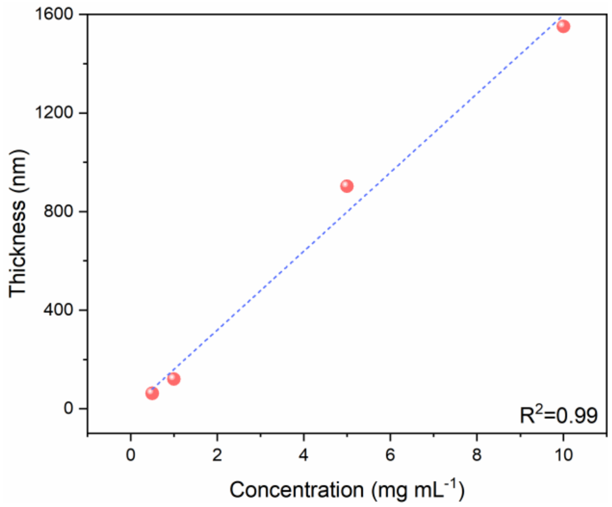

Supplementary Fig. 22. Plot of nanofilm thickness against dispersion concentration of spin-coating. By adjusting the concentration of the GH-TMC dispersion, the film thickness could be controlled and fine-tuned, ranging from the ultra-thin 20 nm down to micrometer-scale level.

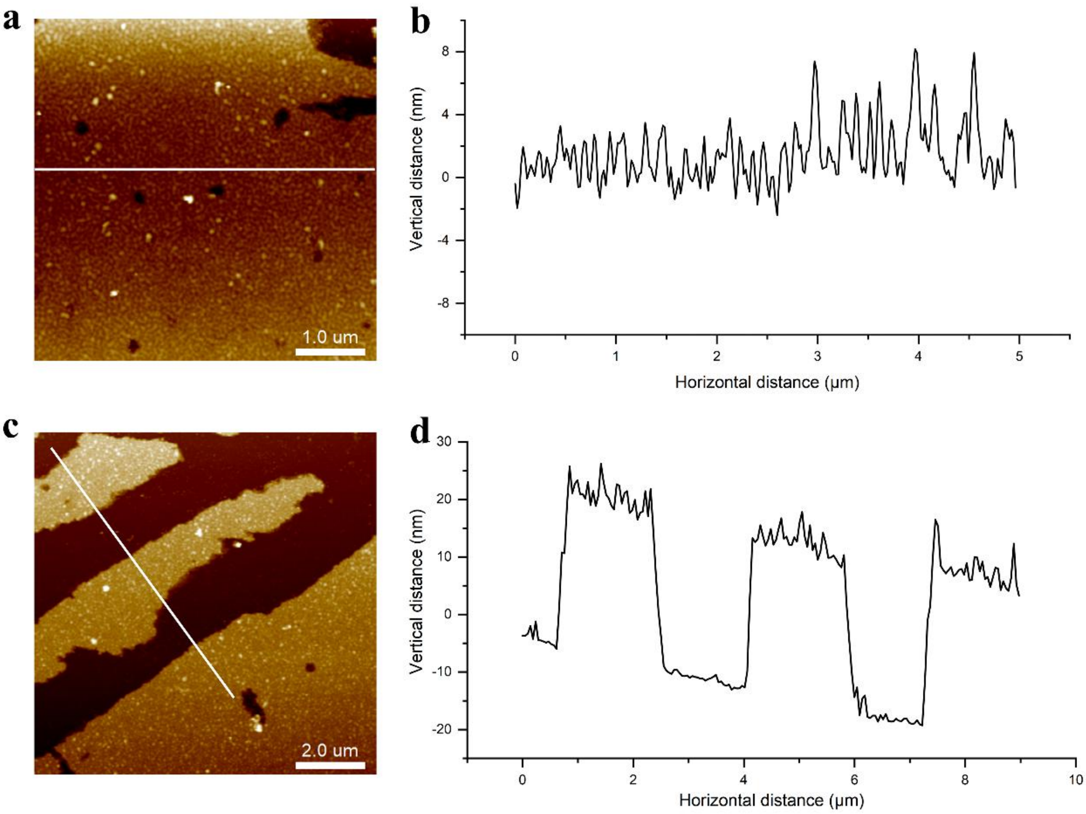

Supplementary Fig. 23. AFM topology of ultra-thin spin-coated GH-TMC films. **a**, AFM topology of ultra-thin GH-TMC film. **b**, Corresponding height profile along the white line in (**a**). **c**, AFM topology of ultra-thin GH-TMC film transferred to mica. **d**, The height profile of line along the white line in (**c**). Note that although some partial breakage may occur during transfer, the film retains continuity and flatness across the substrate.

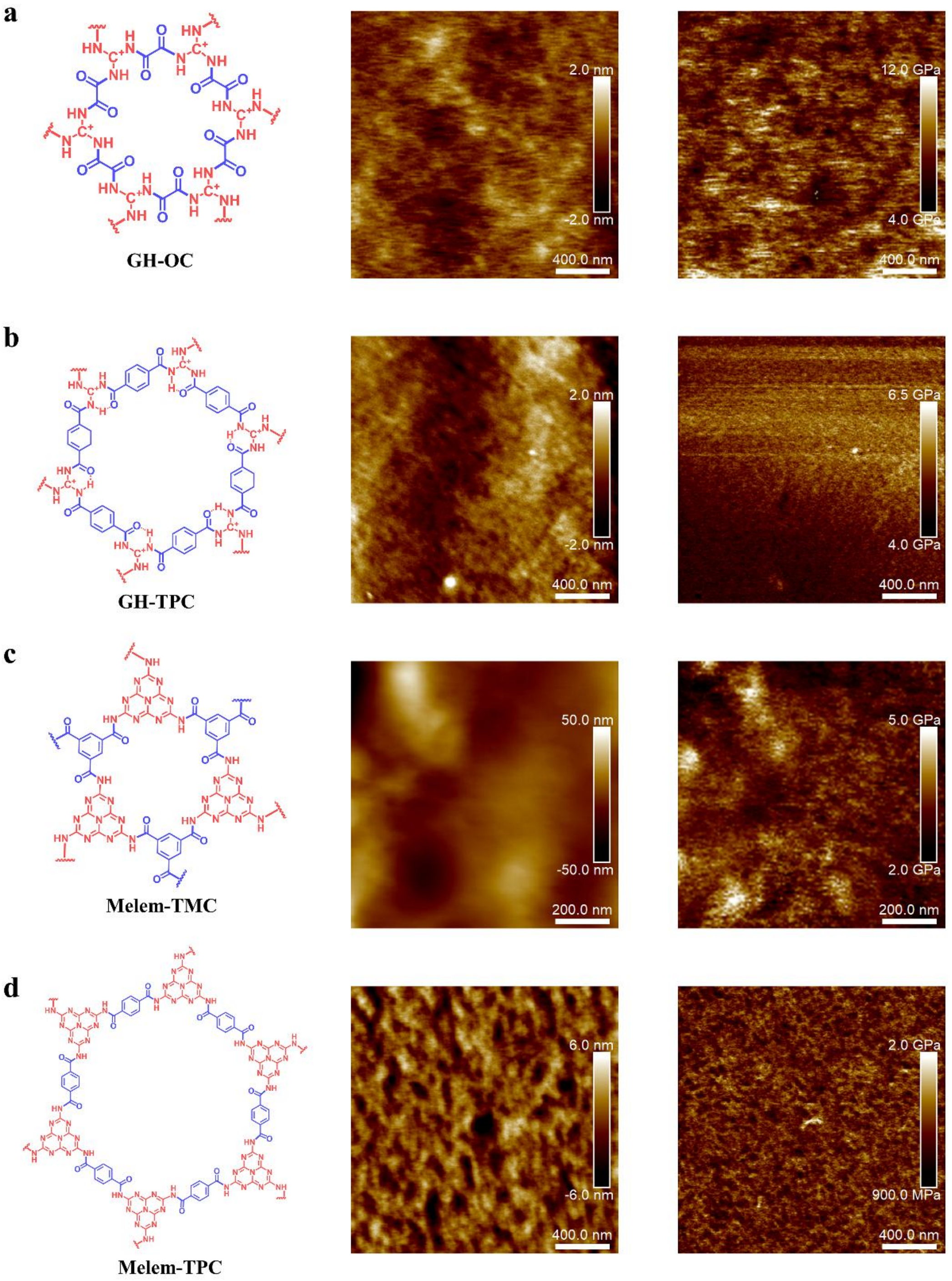

Supplementary Fig. 24. Young's modulus distribution of a series of 2D polymer films. a, AFM image and Young's modulus distribution of GH-OC film. **b**, AFM image and Young's modulus distribution of GH-TPC film. **c**, AFM image and Young's modulus distribution of Melem-TMC film. **d**, AFM image and Young's modulus distribution of Melem-TPC film. All the spin-coated films were tested by AFM in PF-QNM mode and the force provided was maintained at 50 nN.

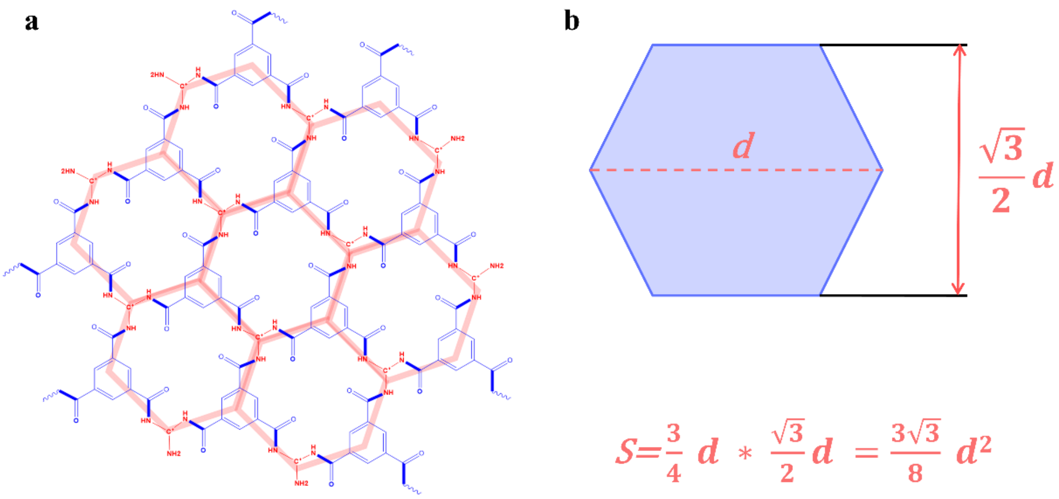

Supplementary Fig. 25. Schematic diagram of the GH-TMC structure. a, The overall structural diagram of each layer of GH-TMC. **b,** Schematic diagram of the structural unit area of 2D polymer. From the mass spectrometry analysis in Supplementary Fig. 2, it was observed that the mass-to-charge ratio of the M4 fragment is consistent with the theoretical value of the proposed hexagonal structure. This confirms that the chemometrics assembly is consistent with the hexamer unit. Solid-state ^{13}C nuclear magnetic resonance (Supplementary Fig. 3) revealed the through-bond correlation characteristics of the structural connection mode and the related chemical environments of different C atoms, which is consistent with the amide bond connection structure we envisioned. Furthermore, we verified the existence of amide bonds and guanidine ions through XPS and FTIR spectra (Supplementary Fig. 4 and 9), which were consistent with the hexagonal motifs. Each TMC is connected to three GH units through amide bonds, forming a cyclic hexamer. The overall structural diagram of GH-TMC is shown in Supplementary Fig. 25a. Each layer of GH-TMC is composed of hexagonal basic structural units. Therefore, we regard the basic structural unit of GH-TMC as a regular hexagonal structure. Similarly, the structural units of all synthesized 2D polymers are approximately regular hexagonal structures, so the area of their structural units can be calculated using the area formula of a regular hexagon (Supplementary Fig. 25b). When calculating the structural model of Young's modulus, it is all regarded as a regular hexagonal structure.

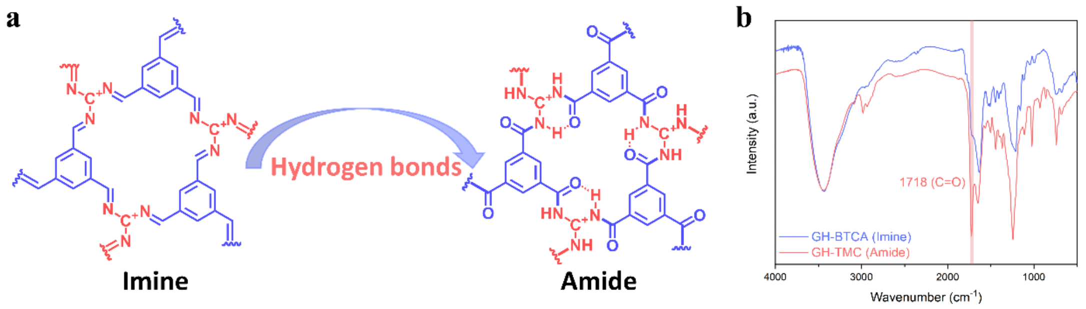

Supplementary Fig. 26. FTIR spectra of amide and imide 2D polymers. **a**, The structural units of GH-TMC (amide) and GH-BTCA (imine). **b**, FTIR spectra of GH-TMC (amide) and GH-BTCA (imide). The disappearance of the amide peak at 1718 cm^{-1} and the formation of the imine peak at 1621 cm^{-1} in the FTIR spectrum of GH-BTCA indicate that the imine is used as the connection node. This modification prevents GH-BTCA molecules from participating in H-bonding interactions.

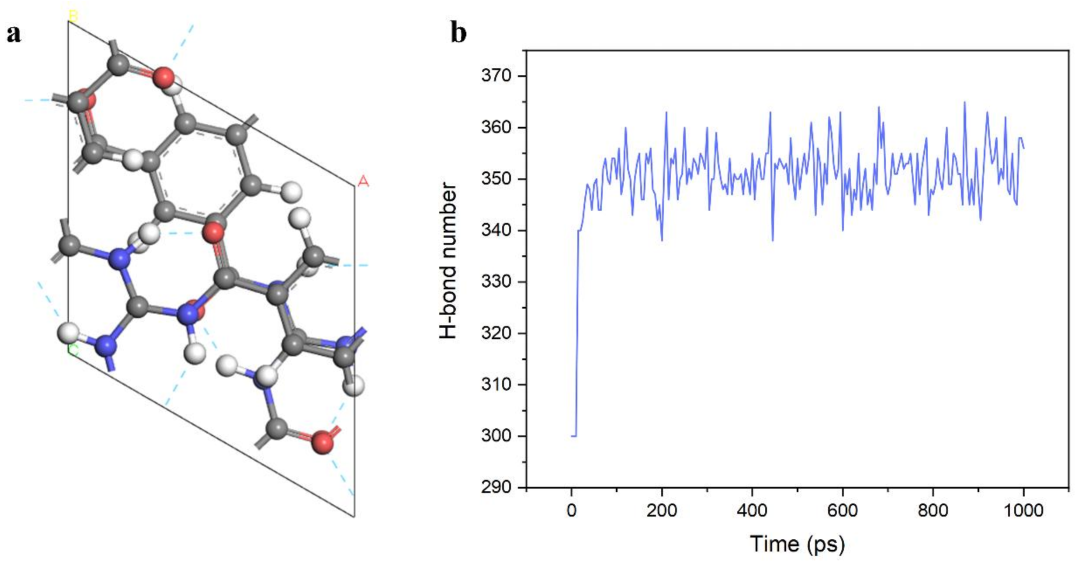

Supplementary Fig. 27. MD simulates the dynamic changes of H-bonds in GH-TMC. a, Simulated interlaced H-bonds at the edges of GH-TMC nanosheets. **b,** MD simulates the dynamic changes of H-bonds in GH-TMC. The H-bond density in 2D polyaramid films plays a critical role in determining their mechanical strength and stability. 1) In-plane strength: In polyaramid films, in-plane H-bonds form between functional groups (amide groups) in the polymer backbone. Higher density of H-bonds means that more strong, directional interactions are available to resist mechanical deformation, which improves the in-plane tensile strength of the membrane. 2) Interlayer strength: 2D polyaramid films can be structured as layered materials, where the interlayer interactions are crucial for maintaining integrity. H-bonding between layers at the edges enhances interlayer adhesion and improve the overall mechanical strength, reducing the likelihood of delamination or layer slippage under stress, which confers the elasticity of the film. To analysis the effect of the H-bonds within the 2D structure, we choose forcite module to do geometry optimization and dynamic molecular computations. To gain a better understanding of the H-bonds interaction, we firstly expanded the unit cell into a 5 * 5 * 2 supercell. The relaxation of all the atoms was performed with the convergence tolerance was set to 2.00 * 10^{-5} and 0.001 kcal mol^{-1} for maximum force. Van der Waals interactions were calculated using atom-based summation, and electrostatic interactions were treated using Ewald summation with a cutoff distance of 18.5 Å and a buffer width of 0.5 Å. COMPASS II force field was

applied here as it is suitable for our system. MD were carried out after geometry optimization in NPT ensembles (N: number of particles; P: pressure; T: temperature) at 0.1 MPa and 298.15 K by using the Nosé thermostat. The MD simulations were run for total 1000 ps to analysis the H-bonds. The results of the MD simulation calculation are shown in Supplementary Fig. 27. GH-TMC not only has multiple H-bonds within the plane, but also has interlaced H-bonds at the edges for connecting the nanosheets. In this supercell, the number of H-bonds simulated and calculated is between 340-364. Therefore, we can calculate that the number of H-bonds in the amide bond of GH-TMC is approximately 2.35, which is extremely close to the number of H-bonds we have tested through the established infrared spectroscopy method. In-plane H-bonds can increase bond density, thereby enhancing the stiffness of the covalent network in the plane, that is, increasing the longitudinal modulus. The intermolecular H-bonds at the edges of the nanosheets are endowed with elasticity through reversible breaking/reconstruction.

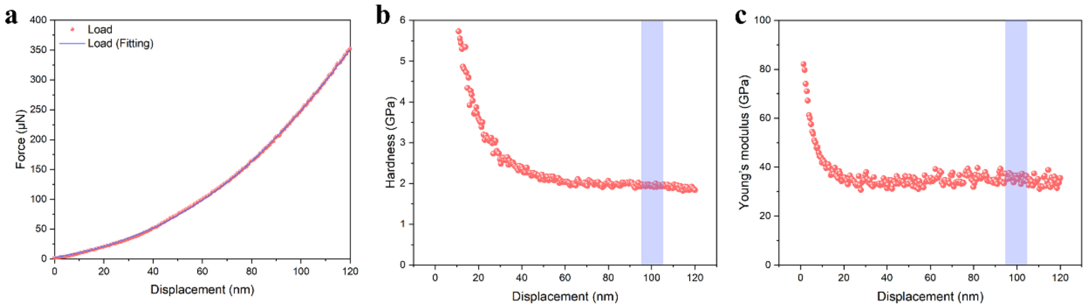

Supplementary Fig. 28. Mechanical properties of GH-TMC. a, Force displacement curve of GH-TMC film. **b**, The average hardness as a function of displacement into the surface. **c**, The average Young's moduli as a function of indentation depth.

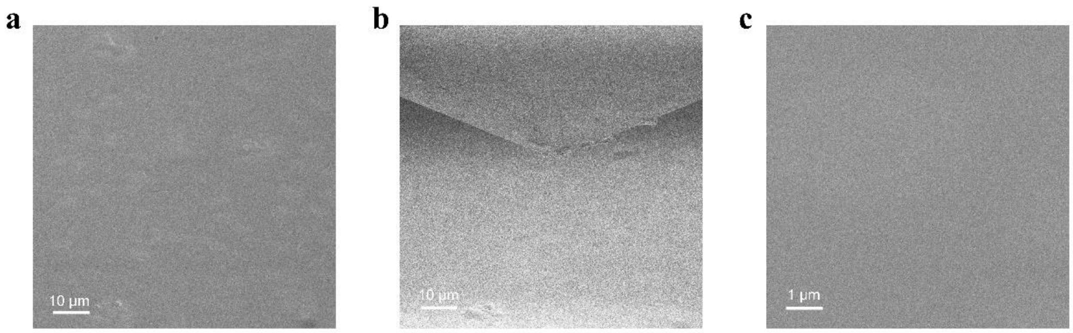

Supplementary Fig. 29. In-situ SEM nanoindentation process at an indentation depth of 120 nm. a, GH-TMC film surface before indentation. **b**, SEM image of GH-TMC film during nanoindentation. **c**, In-situ SEM image of the residual impression left after indentation test. Note that the residual impression was made when the indentation depth was 120 nm. No obvious residual impression after indentation was observed in SEM images, which means that GH-TMC film has good resilience. Scale bars, 10 µm (**a**, **b**), 1 µm (**c**).

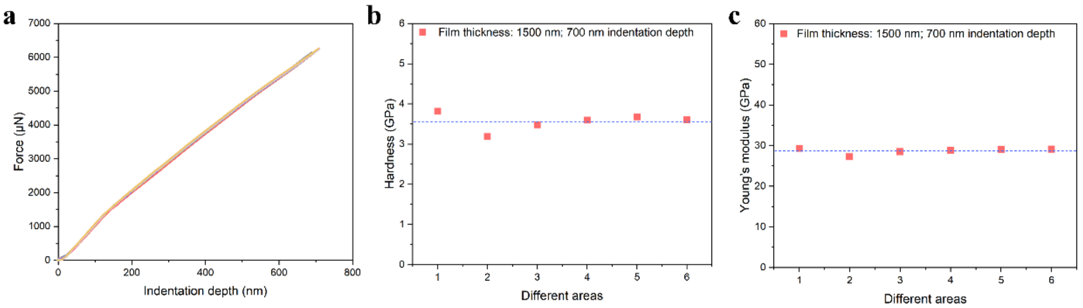

Supplementary Fig. 30. Stiffness and strength of GH-TMC film in different regions. a, Force displacement curves of GH-TMC film in different regions. **b**, Hardness from in-situ SEM nanoindentation at different areas of the GH-TMC film. **c**, Young's modulus from in-situ SEM nanoindentation at different areas of the GH-TMC film. The indentation depth is 700 nm.

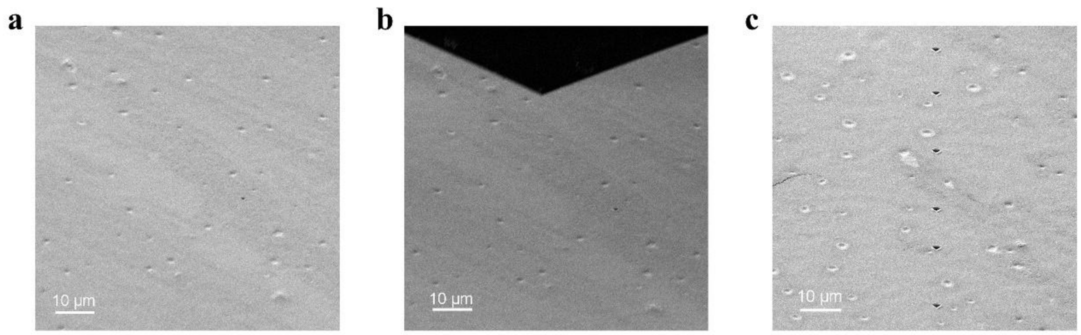

Supplementary Fig. 31. In-situ SEM nanoindentation process at an indentation depth of 700 nm. a, GH-TMC film surface before indentation. **b**, SEM image of GH-TMC film during nanoindentation. **c**, In-situ SEM image of the residual impression left after indentation test. Scale bars, 10 µm (**a**, **b**, **c**). Note that the residual impression was made when the indentation depth was 700 nm.

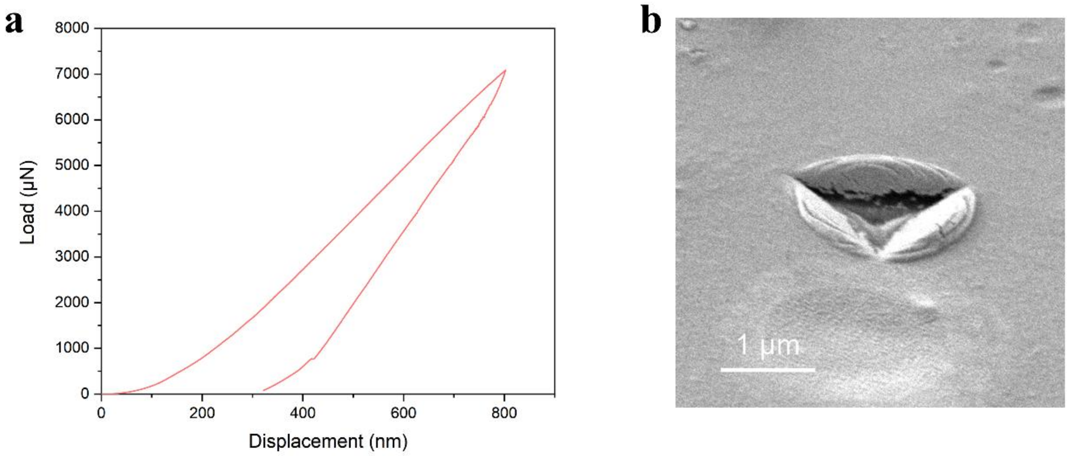

Supplementary Fig. 32. Representative load-displacement curves of GH-TMC film and residual impression at an indentation depth of 700 nm. a, Representative load-displacement curves of GH-TMC film at an indentation depth of 700 nm. **b**, In-situ SEM image of residual impression left after 700 nm indentation depth test. Scale bars, 1 μm (**b**).

Supplementary Table 1. Young's modulus of a series of 2D polymer films.

2D polymer films	Structural unit area (nm^2)	Young's modulus (GPa)
Melem-TPC	7.65	1.29 ± 0.31
Melem-TMC	1.9	3.51 ± 0.68
GH-TPC	3.03	5.14 ± 0.35
GH-OC	1.17	7.83 ± 1.02
GH-BTCA	0.84	17.51 ± 1.38
GH-TMC	0.84	32.77 ± 4.06

Supplementary Table 2. Summary of Young's modulus for GH-TMC film and other countertypes used in this work.

Samples	Young's modulus (GPa)	References
PTFE	0.4	[1]
PP	1.5-2	
PET	2-2.7	
Nylon-6	2-4	
PVC	2.4-4.1	
PS	3-3.5	
$Cu(INA)_2$	0.8 ± 0.3	[4]
MOF-5	2.7	[5]
ZIF-8	3.44 ± 1.42	[6]
$Cu_3(BTC)_2$	3.5 ± 2.5	[4]
ZIF-67	3.79 ± 1.83	[6]
MnDMS	6.5	[7]
Cu(CHDA)	10.9 ± 3.1	[4]
CuBDC	23	[8]
COF-TP-Azo	15.3	[9]
COF-TAPB-DHTA	10.38	[10]
2D polyaramid	12.7 ± 3.8	[11]
Wood	11	[1]
Concrete	17	

Samples	Young's modulus (GPa)	References
Porous carbon	14.5	[12]
Porous silica	13.7-17.5	[13]
**GH-TMC**	**35.612 ± 5.394**	**This work**

[1]. Young's modulus-tensile and yield strength for common materials.

https://www.engineeringtoolbox.com/young-modulus-d_417.html

Supplementary Table 3. Summary of elastic recovery rate (*We*) and hardness (*H*) of GH-TMC film with other countertypes used in this work.

Samples	*H* (GPa)	*We* (%)
PP	0.0953	50.2850
ABS	0.1833	35.3754
POM	0.2372	60.4431
PC	0.1723	45.7245
PMMA	0.2734	44.1508
PS	0.1507	37.0626
PTFE	0.0383	36.6697
PVC	0.2360	25.1475
Al	0.3244	2.9052
Ni	3.4719	15.6680
Cu	1.8519	23.4556
W	7.9011	18.4006
Fe	2.5176	17.4960
AlN	5.7992	48.3341
Calcite	0.2408	27.0812
Dentine	1.0866	32.2461
Enamel	3.1352	31.3082
**GH-TMC**	**2.021 ± 0.615**	**61.7276**

Supplementary Table 4. Comparison between the H^3/E^2 versus H of porous crystalline films.

MOF/COF films	H (GPa)	H^3/E^2 (MPa)	References
$Cu(INA)_2$	0.02 ± 0.01	0.013	[4]
$Cu_3(BTC)_2$	0.17 ± 0.16	0.401	[4]
Cu(CHDA)	0.46 ± 0.14	0.819	[4]
MnDMS	0.4	1.515	[7]
COF-Tp-DPP	0.04 ± 0.005	0.002	[9]
COF-Tp-Azo	0.066 ± 0.002	0.001	[9]
$[(CH_2)_3NH_2][Mn(HCOO)_3]$	0.59 ± 0.05	1.426	[14]
CuBDC	0.448	0.170	[8]
**GH-TMC**	**2.021 ± 0.615**	**6.483 ± 1.382**	**This work**

Supplementary Table 5. Comparison between the H^3/E^2 versus H of polymers.

Polymers	H (GPa)	H^3/E^2 (MPa)	References
PP	0.08 ± 0.053	0.188 ± 0.183	[15]
PA	0.11 ± 0.052	0.294 ± 0.335	[16]
PVA	0152 ± 0.115	0.258 ± 0.183	[17]
PC	0.276 ± 0.104	1.979 ± 1.450	[18]
Epoxy	0.181 ± 0.066	0.742 ± 0.674	[19]
PS	0.266 ± 0.068	0.999 ± 0.689	[20]
PET	0.150	0.247	[21]
PEEK	0.481 ± 0.151	1.497 ± 0.697	[22]
PE	0.072 ± 0.023	0.139 ± 0.102	[23]
PI	0.362 ± 0.088	2.667 ± 1.205	[24]
ABS	0.159 ± 0.025	0.344 ± 0.038	[25]
POM	0.218 ± 0.020	0.800 ± 0.024	[25]
PU	0.013 ± 0.012	0.061 ± 0.036	[26]
PDMS	0.002	0.237	[27]
NBR	0.007 ± 0.002	0.150 ±0.05	[28]
pTA	0.007 ± 0.002	0.042 ± 0.026	[28]
**GH-TMC**	**2.021 ± 0.615**	**6.483 ± 1.382**	**This work**

Supplementary Table 6. Comparison between the H^3/E^2 versus H of Metals.

Metals	H (GPa)	H^3/E^2 (MPa)	References
Al	0.819 ± 0.455	0.136 ± 0.108	[29]
Al-Cu	1.240 ± 0.170	0.313 ± 0.083	[30]
Ni	2.346 ± 1.126	0.216 ± 0.168	[31]
Ni-Co	1.200	0.057	[31]
Ni-Fe	1.780	0.241	[31]
Ti	2.2920 ± 0.755	0.764 ± 0.593	[32]
Fe	1.824 ± 0.714	0.119 ± 0.087	[33]
Fe-Cr	1.567 ± 0.152	0.091 ± 0.025	[33]
Cu	1.852 ± 0.601	0.493 ± 0.263	[28]
**GH-TMC**	**2.021 ± 0.615**	**6.483 ± 1.382**	**This work**

Supplementary Note 1

The mechanical properties of two-dimensional (2D) GH-TMC films are the result of the combined action of multiple interacting forces. First, we investigated the influence of the size of the ring element on the mechanical properties of 2D polyamide films by preparing a series of films with different rigid ring element structures. The smaller the ring element structure, the stronger the Young's modulus of the 2D film. Therefore, the rigid ring element structure is also one of the factors affecting the mechanical properties of the film. On this basis, we simultaneously prepared GH-TMC films and GH-BTCA films with the same rigid ring unit structure. Since the GH-TMC films are connected by amide bonds, they possess triple H-bonds. In the mechanical property tests of films, the Young's modulus of GH-TMC films with H-bond networks is significantly higher than that of GH-BTCA films without H-bonds. Therefore, we propose that the H-bond network can enhance the Young's modulus of 2D films, thereby improving their overall mechanical properties. In addition, as shown in Fig. 3g, not only the size of the rigid structural units constituting the film and the H-bond network, but also electrostatic interactions, π-π interactions, and different stacking modes will all have an impact on the mechanical properties of the film. Below, we have discussed in detail these factors that affect the mechanical properties of GH-TMC films.

Structural unit

The trend of increasing Young's modulus observed in 2D polyamide materials with smaller structural units is rooted in the following key mechanisms. 1) Enhance the rigidity of covalent networks: Smaller ring units (for example, the six-membered ring

structure in GH-TMC) more effectively constrain molecular motion than larger rings, minimizing bond rotation and deformation under stress. This leads to a higher in-plane stiffness. Smaller units contain more chemical bonds per unit area while enhancing the covalent network's resistance to strain. 2) Optimize the H-bond network: In GH-TMC, adjacent amides in the six-membered ring form three intramolecular H-bonds, creating rigid cyclic units that resist deformation. This contrasts with larger units, in which the geometric constraints of H-bonds are fewer or less. The intermolecular H-bonds at the edges of the nanosheets have achieved reversible breaking/reconstruction, balancing strength and elasticity. Larger units weaken this effect by reducing the ratio of edge area. 3) Synergistic interaction : Smaller units facilitate closer layer spacing and enhance the cohesion of the base plane through π-π interactions. Larger units may weaken this cohesion due to increased porosity or misalignment. Therefore, smaller structural units enhance mechanical strength by maximizing the density of covalent and H-bonds, while optimizing secondary interactions (π-π interactions, electrostatic interactions).

H-bond network

The H-bond density in 2D polyaramid films plays a critical role in determining their mechanical strength and stability. 1) In-plane strength: In polyaramid films, in-plane H-bonds form between functional groups (amide groups) in the polymer backbone. Higher density of H-bonds means that more strong, directional interactions are available to resist mechanical deformation, which improves the in-plane tensile strength of the membrane. 2) Interlayer strength: 2D polyaramid films can be structured as

layered materials, where the interlayer interactions are crucial for maintaining integrity. H-bonding between layers at the edges enhances interlayer adhesion and improve the overall mechanical strength, reducing the likelihood of delamination or layer slippage under stress, which confers the elasticity of the film.

To analysis the effect of the H-bonds within the 2D structure, we choose forcite module to do geometry optimization and dynamic molecular computations. To gain a better understanding of the H-bonds interaction, we firstly expanded the unit cell into a 5 * 5 * 2 supercell. The relaxation of all the atoms was performed with the convergence tolerance was set to $2.00 * 10^{-5}$ and 0.001 kcal mol^{-1} for maximum force. Van der Waals interactions were calculated using atom-based summation, and electrostatic interactions were treated using Ewald summation with a cutoff distance of 18.5 Å and a buffer width of 0.5 Å. COMPASS II force field was applied here as it is suitable for our system. Molecule dynamics (MD) were carried out after geometry optimization in NPT ensembles (N: number of particles; P: pressure; T: temperature) at 0.1 MPa and 298.15 K by using the Nosé thermostat. The MD simulations were run for total 1000 ps to analysis the H-bonds. The results of the MD simulation calculation are shown in Supplementary Fig. 27. GH-TMC not only has multiple H-bonds within the plane, but also has interlaced H-bonds at the edges for connecting the nanosheets. In this supercell, the number of H-bonds simulated and calculated is between 340-364. Therefore, we can calculate that the number of H-bonds in the amide bond of GH-TMC is approximately 2.35, which is extremely close to the number of H-bonds we have tested through the established infrared spectroscopy method.

The enhanced mechanical properties in 2D GH-TMC stem from the synergistic molecular-level H-bond network. Each six-membered ring in GH-TMC has triple H-bonds between adjacent amide groups (N-H···O=C), forming a rigid cyclic structure (Fig. 2a). This can increase the bond density, thereby enhancing the stiffness of the covalent network in the plane. Cyclic constraints and C3-symmetry reduce the rotational entropy of the bond and further increase the Young's modulus. For the intermolecular H-bonds at the edges of nanosheets, when π-π stacking dominates the interlayer cohesion, the 3.38 Å interlayer spacing excludes the H-bonds between the basal planes, confining the intermolecular H-bonds to the edge regions. H-bonds form at the edges of the nanosheets. The reversible H-bond fracture/reconstruction at the edge enables the elastic recovery under the indentation to reach 60%. Among them, the energy dissipation from H-bond fracture alleviates crack propagation and helps to enhance toughness. This double H-bond mechanism, by combining the rigidity of covalent networks and multiple weak interaction forces, endows GH-TMC films with excellent mechanical properties.

Multiple weak interactions

For the mechanical properties of GH-TMC films, multiple weak interaction forces are also one of the important influencing factors. 1) Stacking mode: Due to the repulsive force between positively charged guanidine ions in GH-TMC, the AB stacking mode (Fig. 2b) is adopted, and the interlayer spacing (3.38 Å) is optimized to balance the π-π cohesion and electrostatic repulsion. This structure enhances the in-plane stiffness while achieving elastic recovery through localized H-bonds at the edges. 2) Interlayer

interaction: π-π interaction dominates the cohesion of the basal plane, while the displaced H-bonds at the edges of the nanosheets promote interlayer energy dissipation. 3) Counterion effect: XPS confirmed that Cl^- stabilizes the nanosheets through electrostatic interaction with guanidine, which reduces aggregation and maintains structural integrity during the film formation process. 4) Edge defects: The H-bond breakage/reconstruction at the edge of the nanosheet gives it a 60% elastic recovery as an energy dissipation point, while the uniform stacking of nanosheets minimizes stress concentration, thereby enhancing toughness. The interaction of these factors explains the excellent performance of GH-TMC. High H-bond density and small ring units maximize the covalent network stiffness to achieve a high Young's modulus. AB stacking, π-π interactions and edge H-bonds can achieve recoverable deformation under indentation.

The mechanical properties of 2D GH-TMC films are the result of the combined action of multiple interacting forces. Not only the size of the rigid structural units constituting the film and the H-bond network, but also electrostatic interactions, π-π interactions, and different stacking modes will all have an impact on the mechanical properties of the film. The mechanical properties of the film are the result of the combined effect of multiple factors. It is very difficult to fully quantify the relationship between various interaction forces and the Young's modulus of the film. However, for a certain interaction that plays a dominant role, we can make an approximate estimation based on the experimental data from the tests. For GH-BTCA, which has a similar ring unit structure to GH-TMC and multiple interaction forces other than H-bonds, its

Young's modulus is only 17.51 ± 1.38 GPa. This can be considered that the increased Young's modulus in GH-TMC is only contributed by H-bonds, that is, it accounts for 46% of the total strength contribution. In addition, for GH-OC, it lacks rigid structural units and only has H-bonds and other weak interaction forces. Its Young's modulus is only 7.83 ± 1.02 GPa. Therefore, it can be calculated that the contribution of the smaller rigid structural unit is approximately 30%. The remaining 24% is provided by weak interactions such as π-π, electrostatic, and van der Waals. Although precise and full quantification remains complex, these approximations, in combination with the existing Young's modulus data, strongly demonstrate that H-bonds and smaller rigid structural units are the main load-bearing contribution, and the synergy of π-π, electrostatic forces, and van der Waals forces enhances mechanical strength.

References

1 Shen, J. et al. Fast water transport and molecular sieving through ultrathin ordered conjugated-polymer-framework membranes. *Nat. Mater.* **21**, 1183-1190 (2022).

2 Chowdhury, S. C. & Gillespie, J. W. A molecular dynamics study of the effects of hydrogen bonds on mechanical properties of Kevlar® crystal. *Comp. Mater. Sci.* **148**, 286-300 (2018).

3 Deng, S., Ma, D., Zhang, G. & Yang, N. Modulating the thermal conductivity of crystalline nylon by tuning hydrogen bonds through structure poling. *J. Mater. Chem. A* **9**, 24472-24479 (2021).

4 Van de Voorde, B. et al. Mechanical properties of electrochemically synthesised metal-organic framework thin films. *J. Mater. Chem. C* **1**, 7716-7724 (2013).

5 Bahr, D. F. et al. Erratum: Mechanical properties of cubic zinc carboxylate IRMOF-1 metal-organic framework crystals. *Phys. Rev. B* **76**, 184106 (2007)

6 Krishtab, M. et al. Vapor-deposited zeolitic imidazolate frameworks as gap-filling ultra-low-k dielectrics. *Nat. Commun.* **10**, 3729 (2019).

7 Tan, J.-C., Saines, P. J., Bithell, E. G. & Cheetham, A. K. Hybrid nanosheets of an inorganic-organic framework material: facile synthesis, structure, and elastic properties. *ACS Nano* **6**, 615-621 (2012).

8 Zeng, Z., Flyagina, I. S. & Tan, J.-C. Nanomechanical behavior and interfacial deformation beyond the elastic limit in 2D metal-organic framework nanosheets. *Nanoscale Adv.* **2**, 5181-5191 (2020).

9 Dey, K., Bhunia, S., Sasmal, H. S., Reddy, C. M. & Banerjee, R. Self-assembly-driven nanomechanics in porous covalent organic framework thin films. *J. Am. Chem. Soc.* **143**, 955-963 (2021).

10 Fang, Q. et al. Strong and flaw-insensitive two-dimensional covalent organic frameworks. *Matter* **4**, 1017-1028 (2021).

11 Zeng, Y. et al. Irreversible synthesis of an ultrastrong two-dimensional polymeric material. *Nature* **602**, 91-95 (2022).

12 Huang, P. et al. On-chip and freestanding elastic carbon films for micro-supercapacitors. *Science* **351**, 691-695 (2016).

13 Fan, H. et al. Modulus-density scaling behaviour and framework architecture of nanoporous self-assembled silicas. *Nat. Mater.* **6**, 418-423 (2007).

14 Li, W. et al. Mechanical tunability via hydrogen bonding in metal-organic frameworks with the perovskite architecture. *J. Am. Chem. Soc.* **136**, 7801-7804 (2014).

15 Bhandari, N. L., Lach, R., Grellmann, W. & Adhikari, R. Depth-dependent indentation microhardness studies of different polymer nanocomposites. *Macromol. Symp.* **315**, 44-51 (2012).

16 Liu, T. X., Phang, I. Y., Shen, L., Chow, S. Y. & Zhang, W. D. Morphology and mechanical properties of multiwalled carbon nanotubes reinforced nylon-6 composites. *Macromolecules* **37**, 7214-7222 (2004).

17 Prasad, K. E., Das, B., Maitra, U., Ramamurty, U. & Rao, C. N. Extraordinary synergy in the mechanical properties of polymer matrix composites reinforced

with 2 nanocarbons. *Proc. Natl. Acad. Sci. U.S.A.* **106**, 13186-13189 (2009).

18 Nagy, P. M. et al. Nanoindentation investigation of carbon nanotube-polymer composites. *Internet Electron. J. Mol. Des.* **5**, 135-143 (2006).

19 dos Santos, M. N. et al. Thermal and mechanical properties of a nanocomposite of a photocurable epoxy-acrylate resin and multiwalled carbon nanotubes. *Mater. Sci. Eng. A* **528**, 4318-4324 (2011).

20 Remili, C., Kaci, M., Benhamida, A., Bruzaud, S. & Grohens, Y. The effects of reprocessing cycles on the structure and properties of polystyrene/Cloisite15A nanocomposites. *Polym. Degrad. Stabil.* **96**, 1489-1496 (2011).

21 Chen, J. et al. Nanomechanical properties of graphene on poly (ethylene terephthalate) substrate. *Carbon* **55**, 144-150 (2013).

22 Molazemhosseini, A., Tourani, H., Naimi-Jamal, M. R. & Khavandi, A. Nanoindentation and nanoscratching responses of PEEK based hybrid composites reinforced with short carbon fibers and nano-silica. *Polym. Test.* **32**, 525-534 (2013).

23 Bakshi, S. R., Balani, K., Laha, T., Tercero, J. & Agarwal, A. The nanomechanical and nanoscratch properties of MWNT-reinforced ultrahigh-molecular-weight polyethylene coatings. *JOM* **59**, 50-53 (2007).

24 Wahab, M. A., Mya, K. Y. & He, C. Synthesis, morphology, and properties of hydroxyl terminated-POSS/polyimide low-k nanocomposite films. *J. Polym. Sci. A Polym. Chem.* **46**, 5887-5896 (2008).

25 Maries, G. R. E., Bungau, C., Chira, D., Costea, T. & Mosteanu, D.-E. Study on

the influence of the grind percentage over the surface hardness and modulus of elasticity of parts made of ABS, P6.6 and POM through nanoindentation. *Mater. Plast.* **56**, 65-70 (2019).

26 Yusoh, K., Jin, J. & Song, M. Subsurface mechanical properties of polyurethane/organoclay nanocomposite thin films studied by nanoindentation. *Prog. Org. Coat.* **67**, 220-224 (2010).

27 Wu, C.-L., Lin, H.-C., Hsu, J.-S., Yip, M.-C. & Fang, W. Static and dynamic mechanical properties of polydimethylsiloxane/carbon nanotube nanocomposites. *Thin Solid Films* **517**, 4895-4901 (2009).

28 Fang, W. et al. Organic-inorganic covalent-ionic molecules for elastic ceramic plastic. *Nature* **619**, 293-299 (2023).

29 Ram, H. R. A., Koppad, P. G. & Kashyap, K. T. Nanoindentation studies on MWCNT/aluminum alloy 6061 nanocomposites. *Mater. Sci. Eng. A* **559**, 920-923 (2013).

30 Koch, S., Abad, M. D., Renhart, S., Antrekowitsch, H. & Hosemann, P. A high temperature nanoindentation study of Al-Cu wrought alloy. *Mater. Sci. Eng. A* **644**, 218-224 (2015).

31 Yang, L. et al. Deformation mechanisms in single crystal Ni-based concentrated solid solution alloys by nanoindentation. *Mater. Sci. Eng.* **856**, 143685 (2022).

32 Mante, F. K., Baran, G. R. & Lucas, B. Nanoindentation studies of titanium single crystals. *Biomaterials* **20**, 1051-1055 (1999).

33 Zhu, P., Zhao, Y., Agarwal, S., Henry, J. & Zinkle, S. J. Toward accurate

evaluation of bulk hardness from nanoindentation testing at low indent depths. *Mater. Des.* **213**, 110317 (2022).
