# Supplementary material for: Manipulating mechanical strength of isoreticular two-dimensional polyamide materials via multiple interactions
Source: Nat Commun. 2025 Nov 23;16:11580. doi: 10.1038/s41467-025-66696-7 (PMC12748856; doi:10.1038/s41467-025-66696-7)
Supplement: Supplementary file 2 — Description Of Additional Supplementary File [file 41467_2025_66696_MOESM2_ESM.pdf]

Description of Additional supplementary files

Supplementary Movie 1: Hydrogen bond molecular dynamics simulation movie

Description: This Supplementary Movie 1 is a nanoindentation test movie observed through in-situ SEM nanoindentation when the indentation depth is 120 nm.

Supplementary Movie 2: In-situ nanoindentation movie

Description: This video shows a 5 * 5 * 2 supercell running MD simulation over a total of 1000 ps to analyze the H-bond dynamics.
